# Intervention planning for a digital intervention for self-management of hypertension: a theory-, evidence- and person-based approach

**DOI:** 10.1186/s13012-017-0553-4

**Published:** 2017-02-23

**Authors:** Rebecca Band, Katherine Bradbury, Katherine Morton, Carl May, Susan Michie, Frances S. Mair, Elizabeth Murray, Richard J. McManus, Paul Little, Lucy Yardley

**Affiliations:** 1 Centre for Clincial and Community Applications of Health Psychology, University of Southampton, Shackleton Building, Highfield Campus, Southampton, SO17 1BJ UK; 2 Faculty of Health Sciences, University of Southampton, Southampton, UK; 3 UCL Centre for Behaviour Change, Department of Clinical, Educational and Health Psychology, University College London, 1-19 Torrington Place, London, WC1E 7HB UK; 4 Institute of Health and Wellbeing, University of Glasgow, Glasgow, G12 9LX Scotland; 5 Research Department of Primary Care and Population Health, University College London, Rowland Hill Street, London, NW3 2PF UK; 6 Nuffield Department of Primary Care Health Sciences, University of Oxford, Oxford, UK; 7 Primary Care and Population Sciences, Faculty of Medicine, University of Southampton, Southampton, UK

**Keywords:** Intervention planning, Theoretical modelling, Methodological study, Hypertension, Blood pressure, Self-monitoring, Self-management

## Abstract

**Background:**

This paper describes the intervention planning process for the Home and Online Management and Evaluation of Blood Pressure (HOME BP), a digital intervention to promote hypertension self-management. It illustrates how a Person-Based Approach can be integrated with theory- and evidence-based approaches. The Person-Based Approach to intervention development emphasises the use of qualitative research to ensure that the intervention is acceptable, persuasive, engaging and easy to implement.

**Methods:**

Our intervention planning process comprised two parallel, integrated work streams, which combined theory-, evidence- and person-based elements. The first work stream involved collating evidence from a mixed methods feasibility study, a systematic review and a synthesis of qualitative research. This evidence was analysed to identify likely barriers and facilitators to uptake and implementation as well as design features that should be incorporated in the HOME BP intervention. The second work stream used three complementary approaches to theoretical modelling: developing brief guiding principles for intervention design, causal modelling to map behaviour change techniques in the intervention onto the Behaviour Change Wheel and Normalisation Process Theory frameworks, and developing a logic model.

**Results:**

The different elements of our integrated approach to intervention planning yielded important, complementary insights into how to design the intervention to maximise acceptability and ease of implementation by both patients and health professionals. From the primary and secondary evidence, we identified key barriers to overcome (such as patient and health professional concerns about side effects of escalating medication) and effective intervention ingredients (such as providing in-person support for making healthy behaviour changes). Our guiding principles highlighted unique design features that could address these issues (such as online reassurance and procedures for managing concerns). Causal modelling ensured that all relevant behavioural determinants had been addressed, and provided a complete description of the intervention. Our logic model linked the hypothesised mechanisms of action of our intervention to existing psychological theory.

**Conclusion:**

Our integrated approach to intervention development, combining theory-, evidence- and person-based approaches, increased the clarity, comprehensiveness and confidence of our theoretical modelling and enabled us to ground our intervention in an in-depth understanding of the barriers and facilitators most relevant to this specific intervention and user population.

**Electronic supplementary material:**

The online version of this article (doi:10.1186/s13012-017-0553-4) contains supplementary material, which is available to authorized users.

## Background

Elevated blood pressure (BP) is currently the highest risk factor for global disease burden, accounting for 7% of global disability-adjusted life years (DALYs) [1] due to the increased risk of cardiovascular diseases, such as heart attack or stroke [2]. The Health Survey for England (2012) identified that approximately 30% of the adult population have hypertension [3]. It has been estimated that a 10 mmHg reduction in BP could lead to a 41% reduction in stroke and a 22% reduction in CHD [4], and recent findings from the SPRINT trial suggest that further reductions in target BP are beneficial to patient health outcomes [5]. However, both hypertension treatment and control within the UK are currently suboptimal [6], with almost 20% of the variance in BP control accounted for by ‘clinical inertia’—clinician failure to intensify treatment when necessary [7, 8]. Inadequate management of hypertension may also result from lack of patient engagement with medication and other self-management behaviours [9, 10]. Interventions using patient self-monitoring of BP as the basis for more rapid medication escalation have been shown to be an effective method to reduce BP levels [9, 11–16]. Interventions combining intensive support from a variety of sources including medication titration, patient education and pharmacist support appear to be the most effective in reducing BP [13, 14, 16].

Digital health interventions offer an opportunity to address the increasing health burden in a potentially cost-effective way [17], by providing automated and remote support for self-management and giving users the benefits of flexible and convenient access and personalised advice and feedback. In the case of hypertension, they might prove a feasible method of supporting patient self-monitoring of blood pressure and healthy behaviour change, integrated with clinician-guided treatment escalation. The aim of the intervention planning process described in this paper was to design a digital intervention (Home and Online Management and Evaluation of Blood Pressure (HOME BP)) for primary care patients with hypertension to support blood pressure self-monitoring, medication titration and healthy behaviour change. The intervention is described in more detail elsewhere [18].

The TASMINH2 trial [11] provided the best existing evidence for an effective UK-based intervention combining patient blood pressure self-monitoring with self-titration of anti-hypertensive medication based on a pre-defined medication escalation protocol in an uncomplicated hypertensive population [11, 12]. The TASMINH2 study was an adaption of an earlier intervention [19]; pragmatic modifications included a more conservative titration procedure defined by the individual clinician to increase acceptance in clinical practice and build patient self-efficacy. The titration procedure adopted in TASMINH2 was therefore selected as an appropriate basis for online adaptation [12], with the addition of a secondary focus on supporting healthy behaviour change, in view of the evidence that this could also be beneficial [20]. However, translation of healthcare interventions into a digital format is associated with a number of development challenges, in particular relating to understanding how intervention elements essential to acceptable and effective implementation can be adapted for automated and remote delivery [21]. Qualitative exploration of patient views suggests a lack of confidence in using digital technology, such as the Internet or apps, to support self-management of blood pressure [22]. Careful intervention planning and development procedures are therefore required to ensure successful implementation of a home-based digital health intervention integrated within the patient’s regular healthcare context [23–25].

The intervention planning and development for the HOME BP study was conducted using a theory-, evidence- and person-based approach [26–28]. The Person-Based Approach to intervention planning advocates generating an in-depth understanding of the intended intervention users through iterative use of qualitative research [27]. When combined with other evidence sources—particularly clinical, intervention developer and public experience [29] and reviews of the relevant quantitative and qualitative literature—this approach grounds intervention planning in a detailed knowledge of the likely barriers and facilitators to implementation. The role of theory in the intervention planning process is varied [30, 31] and includes checking that potentially important drivers of behaviour have not been overlooked, providing a formal method to characterise interventions [32], guiding the process evaluation and identifying potential issues with intervention implementation [24]. The latest Medical Research Council (MRC) guidance advises that the development of complex interventions should systematically draw on the latest evidence and be guided by appropriate theory [33]. However, we argue that complementing these with the Person-Based Approach, an in-depth understanding of the user and the context of the intervention is important for increasing the acceptability and hence likely engagement with and effectiveness of the intervention [34].

This paper provides an illustration of how the Person-Based Approach can be integrated with theory- and evidence-based approaches to intervention planning and development, by outlining the intervention planning process we undertook for HOME BP. HOME BP is a digital intervention integrating patient and healthcare professional intervention components to deliver the anti-hypertensive medication titration procedure and behavioural support for patients undertaking self-management of hypertension. The HOME BP intervention is currently being evaluated in a randomised controlled trial [35]. In this paper, we present the full intervention planning process for the HOME BP intervention, in line with best practice recommendations [33, 36] to allow replication and analysis by other researchers and practitioners.

## Methods and results

**Fig. 1:**
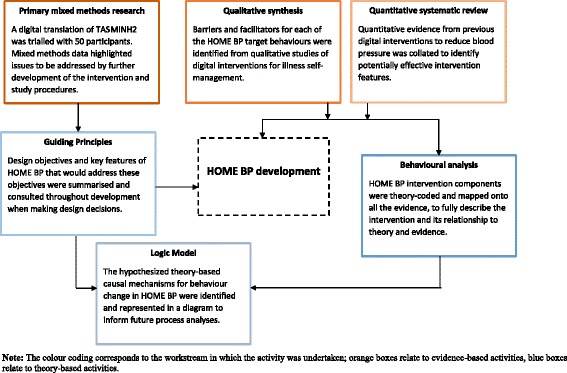
The six elements of intervention planning for HOME BP. Note: The colour coding corresponds to the workstream in which the activity was undertaken; *orange boxes* relate to evidence-based activities, *blue boxes* relate to theory-based activities

In the following sections, we describe the methods and results for each of the six elements of the intervention planning process in HOME BP (see Fig. 1). Work stream 1 comprised three approaches to collating evidence relating to the design and effective implementation of HOME BP, and analysing and synthesising it to identify likely barriers, facilitators and effective design features. Work stream 2 comprised three approaches to theoretical modelling that were used to guide and structure the intervention design, description and evaluation.

These two streams of work, and also the intervention development and evaluation, were carried out iteratively and in parallel, with the different aspects contributing to one another. The intervention planning documents were therefore updated throughout the intervention development process to incorporate and synthesise evidence and qualitative development work as it emerged. Monthly intervention planning and development meetings were conducted, where input on intervention content and proposed procedures was sought from a variety of sources including experts in hypertension, primary care clinicians, PPI representatives and organisations (Blood Pressure UK) and experts in behavioural science; this expert input was also incorporated into a detailed record of the decision-making process throughout the development of HOME BP.

Each of the following sections briefly describes the method used for each element of intervention planning, together with the output from that method and selected examples of how these outputs fed into the intervention development. The methods for collating evidence are not novel and so are described only briefly below (see Additional file 1 for full details). The findings from the qualitative studies and systematic reviews that informed the intervention planning and development are described in more detail elsewhere [37–40].

### Work stream 1: collating and analysing evidence

#### Primary mixed methods research

##### Purpose

To identify issues relating to the acceptability and feasibility of online implementation of the procedures used in TASMINH2 [11] for blood pressure self-monitoring and titration, supplemented by an Internet-delivered healthy behaviour change programme [41].

##### Methods

A small feasibility study was carried out before planning for HOME BP commenced. The intervention was trialled in 50 participants from 8 practices, and qualitative interviews were carried out with 16 patients and 3 healthcare professionals. Debriefing focus groups were also carried out with a further 8 health professionals. Open-ended questions elicited views of the intervention, focusing particularly on issues of acceptability and feasibility [42] (see Table 1).

**Table 1:** Additional information about the primary mixed methods research

	Practices	Patients	Healthcare professionals
Recruitment route	CRN Wessex	Practice mail-out	Practice manager
*n*	8	50	16
Qualitative interview topics	–	Thoughts about the website, experiences of monitoring, entering BP readings into the website, BP reading feedback, experiences of medication change processes, experiences of behavioural support, lifestyle changes	Thoughts about the website, experiences of the study and procedures, experiences of supporting patients (in relation to medication changes or lifestyle change), communication between healthcare professionals involved in the study, how the procedure fit with current working practices

##### Results

Key issues arising from the feasibility study are summarised in Table 2, which also explains how HOME BP was designed to address these issues. A crucial insight from this stage of the intervention planning was that translating the TASMINH2 intervention into an effective Internet-delivered intervention was not simply a matter of transferring written materials online. It proved difficult for primary care staff to implement the intervention independently, without any input from the research team. To encourage primary care staff and patients to adhere to the titration protocol, it was necessary to put in place easily implemented online procedures, supported by safety checks and reassurance (e.g. about side effects and medical supervision), to ensure that both patients and medication prescribers would feel motivated and confident to undertake titration without a consultation.

**Table 2:** Key feedback from feasibility study focus groups and interviews (patients and health professionals) and how this informed intervention design in HOME BP

Issue identified by qualitative research	HOME BP design feature addressing this issue
Patients did not regard hypertension as a serious problem requiring active management.	A motivational quiz was added to the first website session to highlight the potential serious consequences of uncontrolled hypertension.
Patients were happy to self-monitor their blood pressure, but most felt they had already made sufficient healthy behaviour changes and were not highly motivated to undertake further behaviour changes to manage hypertension.	Since medication titration is more effective than behavioural management of hypertension, the HOME BP intervention was designed so that all patients undertook titration as their primary aim but were encouraged to also undertake behaviour changes to avoid future medication increases.
The medication titration procedures were not implemented as planned, because: a) receptionists were unaware of the automated procedure and so booked patients for a GP appointment when they contacted the practice with raised blood pressure; b) prescribers forgot or had missed out on the training and were not picking up their reminder emails, so just proceeded with usual care rather than following the titration protocol.	The HOME BP intervention was designed so that: a) the prescriber was emailed directly to make required titrations by issuing a prescription (avoiding the need for a consultation); b) the online and offline procedures were re-designed (with central monitoring) and a practice lead designated to ensure that prescribers were aware of the intervention, had completed training and were accessing emails from the intervention.
Some patients were not receiving nurse support. Some nurses did not recall their training and were unaware that they needed to check the study email account, hence were not picking up reminder emails from the automated intervention or emails from patients requesting support.	The study procedures were re-designed so that nurses had to complete online training before they could recruit patients and could re-access this training at any point during the intervention. Emails prompting nurses to provide support were sent to their personal email account and to a general study account which the practice manager took responsibility for overseeing.

#### Qualitative synthesis of relevant literature

##### Purpose

To collate evidence from qualitative studies examining patient, healthcare professional and other stakeholder perspectives and experiences of using telemedicine or digital interventions to support self-management in hypertension, asthma and other similar long-term health conditions.

##### Methods

An initial rapid scoping review of the literature was necessary to ensure that the evidence identified could be quickly incorporated into the initial intervention planning and development phases [43] (see Additional file 2). To inform the intervention planning, data extraction comprised a description of the intervention components (where available), evidence of facilitators and barriers with respect to using digital health interventions and other findings reported within the paper (see Additional file 3 for a four-page excerpt from the extensive data extraction table). Thematic analysis was conducted on the extracted data; findings were organised around facilitators and barriers relating to each theme (see Additional file 4). Additional information regarding how facilitators were (or could be) used and the ways in which barriers were (or could be) addressed was also recorded.

##### Results

Five key themes emerged from the initial qualitative synthesis, relating to patient experiences of self-management using digital health interventions, blood pressure self-monitoring, medication adherence and intensification, and healthcare professional experiences of digital interventions, and confidence in online systems. Additional file 4 provides full details of the barriers and facilitators identified by the synthesis relating to both patient and healthcare professional engagement with digital interventions for patient self-management. Selected examples of how this evidence informed intervention planning are provided below.

**Fig. 2:**
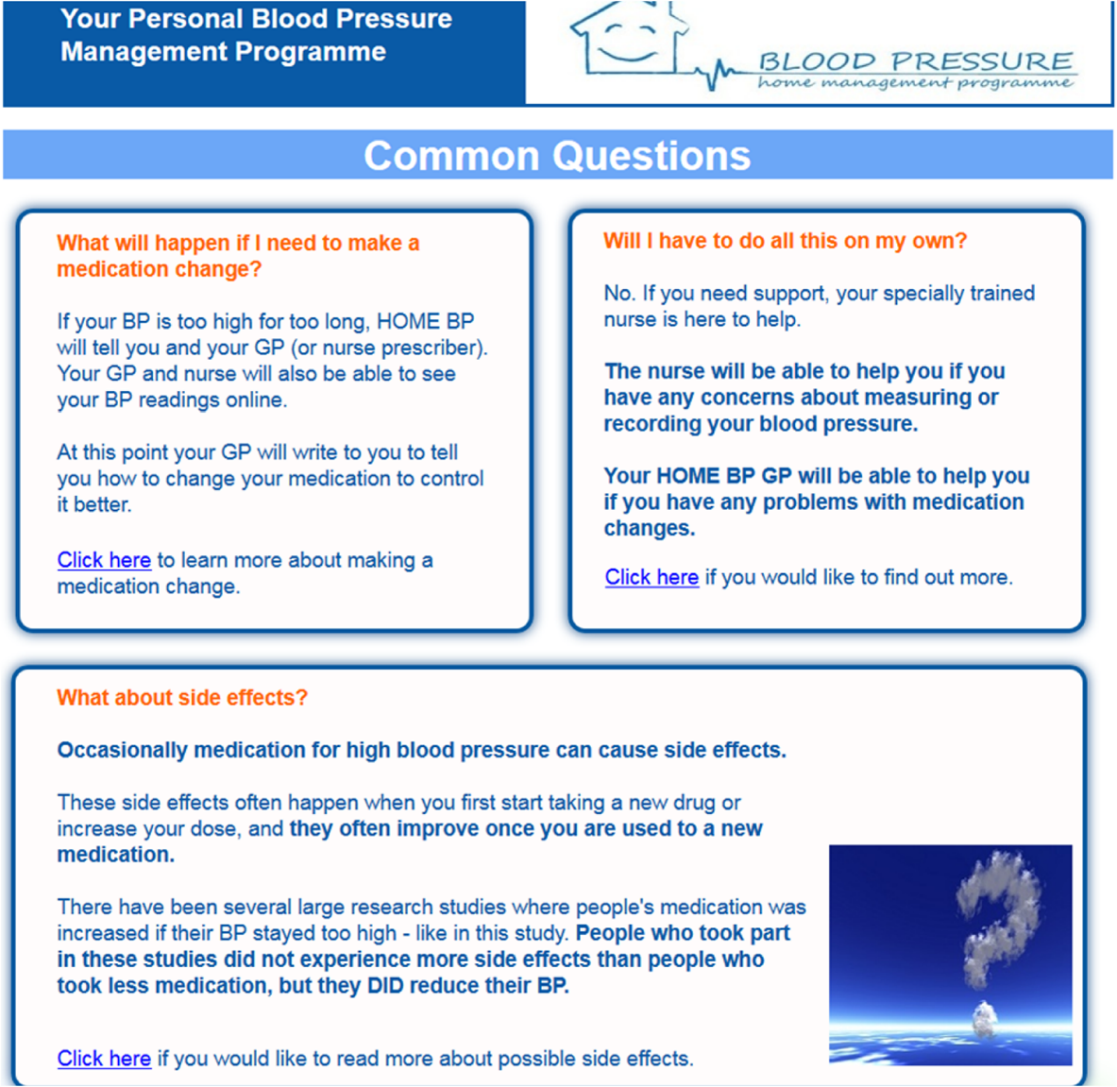
Screenshot from the patient HOME BP intervention version addressing patient concerns regarding the side effects of anti-hypertensive medication

The evidence suggested that healthcare professional confidence in the system, particularly with reference to the reliability and accuracy of readings, was an important factor to consider. We therefore emphasised in the health professional training materials that home blood pressure readings were more accurate than clinic readings as the basis for clinical decision-making and that the titration procedures were based on current gold standard procedures for hypertension control [11]. We also used the qualitative evidence to provide further support for some HOME BP design decisions suggested by our primary qualitative research. For example, the qualitative literature confirmed that the information provided in HOME BP would need to be motivating, providing strong evidence for the benefit of titrating medications, and addressing potential concerns about unwanted side effects. A screenshot illustrating how this was implemented within the patient version of HOME BP is provided in Fig. 2. The in-depth meta-synthesis conducted subsequently is published elsewhere [39].

#### Quantitative systematic review

##### Purpose

To collate evidence from quantitative studies of digital interventions to reduce blood pressure and intervention features associated with better outcomes.

##### Methods

A systematic review was conducted to identify digital interventions whose primary or secondary outcomes included reduction in blood pressure [38]. For the purposes of the intervention planning and development process, relevant papers that were excluded against review criteria (for example, non-interactive telemedicine interventions) were also used to inform HOME BP planning. Following a similar approach to that reported by Baxter and colleagues [44], we also considered non-trial sources such as systematic reviews, meta-analyses and any other relevant papers identified by the search and the research team. Detailed information about the intervention components and study procedures was extracted (where relevant) and tabulated, together with reported effectiveness and cost-effectiveness. Selected excerpts from these extensive extraction tables are provided in Additional file 5. Evidence relevant to intervention component design features was incorporated into the intervention planning tables, and cross-referenced in the record of decision-making (see example below), and also fed immediately into the development of HOME BP. Through consultation and discussion, input was also obtained from all members of the development and planning group (which included primary care clinicians, experts in behavioural science, patient representatives and experts in hypertension) regarding the essential level of support required to increase adherence without increasing face-to-face consultation, considering the feasibility requirements for potential future NHS implementation during monthly meetings.

##### Results

**Fig. 3:**
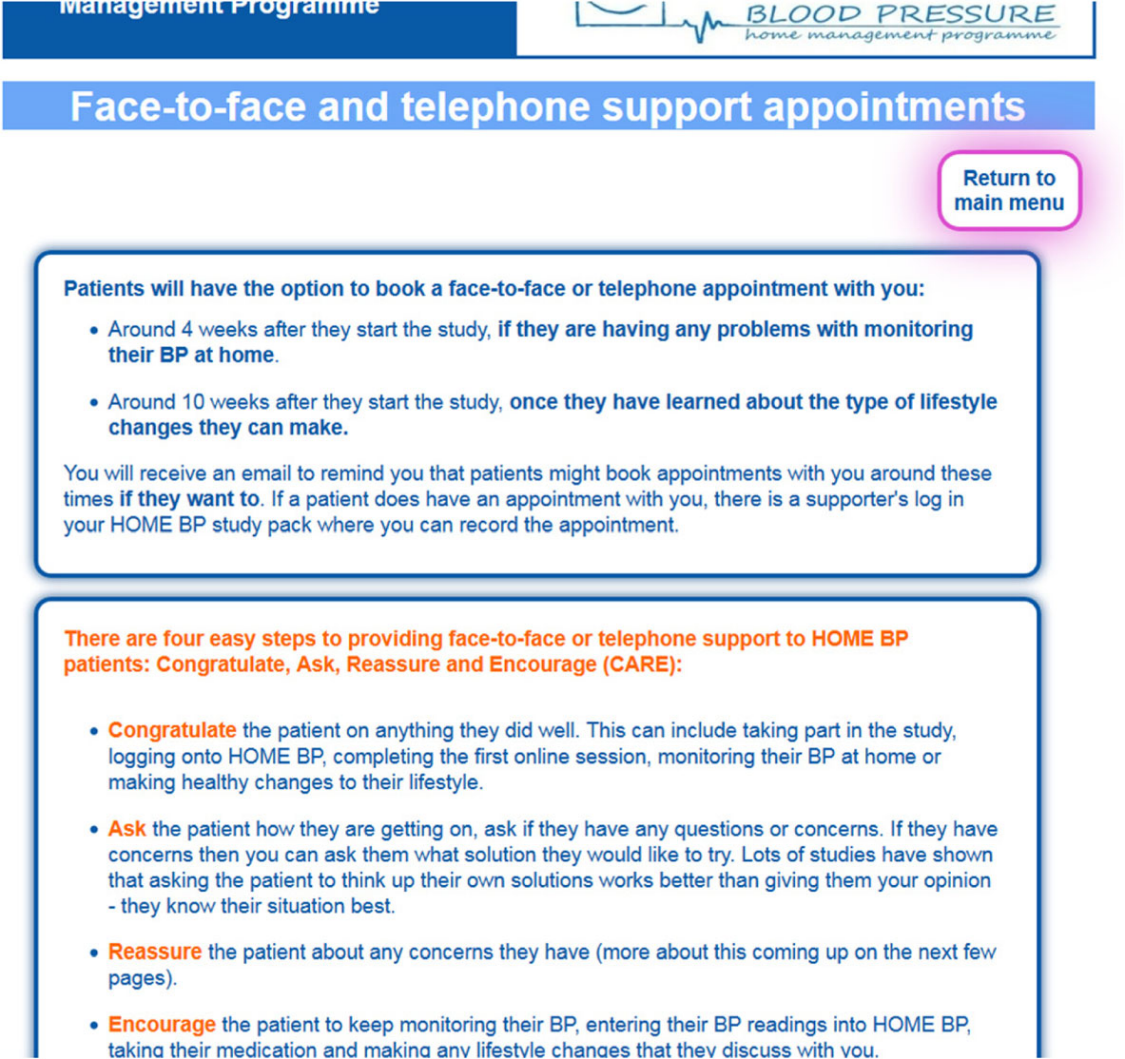
Screenshot from the supporter intervention pages outlining the CARE approach to behavioural support within HOME BP

The review confirmed that self-management interventions can lead to reductions in blood pressure [38], and additional important design features relevant for HOME BP were identified. For example, a key issue arising from the quantitative literature was that previous interventions reporting efficacious reductions in participant blood pressure had used relatively intensive behavioural support, providing behavioural support as frequently as every 2 weeks until blood pressure was controlled [13, 14]. Whilst the evidence suggested that support was a beneficial addition to self-monitoring, meta-analyses suggested the optimum level of support is unclear [16]. Moreover, the planning and development of HOME BP had to balance the potential benefits of health professional support with what would be feasible and cost-effective to offer within a UK primary care context. As a result, it was decided that face-to-face support would be offered for the first week of self-monitoring and after the initiation of behaviour changes, as these are key times within the intervention when patients are likely to require additional support. It was decided that regular support (every 4 weeks) would be provided to the patient by email and that the patient would be able to request additional support at any time through the HOME BP programme, restricted to a maximum of six face-to-face or telephone support sessions. An example of the HOME BP healthcare professional pages explaining the procedures and recommended approach to behavioural support provision are provided in Fig. 3.

### Work stream 2: theoretical modelling

#### Guiding principles

##### Purpose

To develop brief ‘guiding principles’, which summarise the key intervention design needs and objectives and the features of the intervention design required to address these.

##### Methods

The first step in creating guiding principles was to state the objectives of the intervention, in terms of key behaviours and outcomes (derived from the research proposal and protocol), and briefly describe relevant aspects of users and their context [27]. Next, we identified key behavioural issues, needs or challenges the intervention must address, drawing on our primary mixed methods research as well as evidence from the qualitative literature review. We then formulated the guiding principles in terms of key intervention design objectives (which were based on the specific important needs, issues and challenges identified by the work stream collating and analysing relevant evidence) and the distinctive design features intended to meet each objective (which were derived from intervention planning, including the evidence base, behavioural analysis and the logic model).

In the development of HOME BP, we began to formulate the guiding principles in the first stages of planning, drawing on prior experiences of the TASMINH, TASMINH2 and TASMIN-SR trials of patient self-monitoring of blood pressure and medication titration [11, 45, 46], our initial feasibility study of implementing these procedures online and our team’s knowledge of relevant literature. The guiding principles were then refined as necessary to incorporate additional needs, objectives and design features as the planning process progressed and wider theory and evidence was considered.

##### Results

The objective of the HOME BP intervention, in terms of outcomes, is to provide cost-effective (hence mainly automated) support to patients with hypertension to improve control of their blood pressure through behaviour change and optimum anti-hypertensive medication titration. In terms of behaviours, this required support for patients to self-monitor their blood pressure and for patients and health professionals to increase their medication if it was not well-controlled. At an early stage of intervention planning, we decided to reduce the emphasis in the intervention on patients also undertaking healthy behaviour change (which was encouraged but not required in HOME BP), as our evidence suggested that most UK primary patients were not motivated to undertake sufficient behaviour change to influence blood pressure [47] and we were concerned that ineffective health promotion attempts could detract from effective implementation of the central target behaviours in HOME BP, which were self-monitoring blood pressure and appropriately escalating medication. Key relevant feedback from target users were that both patients and health professionals had concerns about escalating medication and needed implementation procedures that were perceived as safe, appropriate and very easy to follow. Table 3 presents the guiding principles created to try to achieve these objectives, based on our understanding of the key behavioural issues (described in the table).

**Table 3:** The guiding principles for the development of HOME BP

Intervention design objectives	Key features
To motivate patients and practice staff to undertake medication titration	• Education for patients and staff about benefits of titration and study procedures (e.g. quizzes to promote knowledge, evidence of need and efficacy) • Elements to promote patient and staff self-efficacy and autonomy for undertaking titration (e.g. skill building, emphasising health professional oversight) • Addressing concerns of patients and staff about medication side effects (e.g. encouraging realistic expectations about side effects, providing methods to seek advice on side effects) and of staff about patients’ acceptance of medication titration
To facilitate implementation of medication titration by patients and practice staff	• Carefully designed automation of practice-patient interaction to make implementation of titration procedures as easy and well-controlled as possible • In-built procedures to manage patient or staff concerns or objections to titration
Easy and low cost to implement the protocol	• Limiting the study co-ordinator role • Online training • No passwords for healthcare practitioner log on to ensure easy access to procedures, training and documentation • Prioritising medication titration as the key behaviour in reducing hypertension • Providing optional (and flexible) support at the most crucial time points

#### Behavioural analysis

##### Purpose

To use behaviour change theory to code the HOME BP intervention content and map it onto the evidence derived from work stream 1.

##### Methods

Our planning process was initially data-driven, in the sense that it was based on the evidence identified in the first work stream. The behavioural analysis tables recorded the four key patient target behaviours (engaging with the intervention, home blood pressure self-monitoring, medication adherence and titration, and healthy behaviour change) as well as the subsidiary behaviours necessary to enact the key target behaviours. For example, in order to achieve adherence to a new medication, patients must accept the recommended change without a face-to-face GP consultation, collect the new prescription and medication, and then begin the new medication regime. The healthcare professional (HCP) intervention planning tables included three key target behaviours: engaging with the intervention, enacting medication titration procedures and behavioural support provision. The corresponding intervention component designed to elicit the target behaviour was also recorded.

To undertake the behavioural analysis, we coded the intervention content using the Behaviour Change Wheel (BCW) [32] and Normalisation Process Theory (NPT) [48]. The BCW is a theoretical framework used for characterising interventions; it allows researchers to analyse the likely source of behaviour and link this to the intervention function and the fine-grained behaviour change techniques being utilised [32]; using NPT allowed us to characterise HOME BP use within the patient and healthcare professional context, by considering, for example, how individuals would incorporate self-monitoring into daily life, as well as implementation at an organisational level [24]. Using the COM-B (‘capability’, ‘opportunity’, ‘motivation’ and ‘behaviour’) model, the source of each target behaviour and specific intervention functions were first coded onto the BCW [32]. Each behaviour change technique used in the intervention was then mapped using the 93-item Behaviour Change Technique taxonomy v1 [49]. Finally, potential determinants of change (i.e. mechanisms of implementation) outlined within the NPT framework were applied to each of the target behaviours, and the relevant NPT mechanism and construct recorded within the tables. Subsequently, we examined each of our theoretical frameworks to check for any potential useful additional intervention components or behavioural targets that had not been identified through the evidence- and person-based approaches. For this analysis, we defined what each specific BCW and NPT construct would mean in the context of the HOME BP intervention (for example, skill set workability would refer to participants having the necessary skills to carry out the target behaviours) and checked for corresponding HOME BP intervention components (i.e. training for home self-monitoring of blood pressure).

##### Results

The HOME BP intervention planning tables consisted of 11 pages and included both the patient and health professional intervention components. The full HOME BP planning tables are presented in Additional file 6 and include all of the behavioural determinants and behaviour change techniques included in HOME BP.

Our theoretical analysis of the determinants of blood pressure self-monitoring, using the BCW, suggested that HOME BP was targeting various behavioural sources, specifically physical and social opportunity, reflective motivation and psychological capability. Our theoretical analysis of the intervention components designed to promote self-monitoring identified that HOME BP employed five different intervention functions from the COM-B model [32] (education, persuasion, training, enablement, environmental restructuring) using ten different behaviour change techniques (see Additional file 6 for where and how these were used). Behavioural analysis of our intervention components was also undertaken using the NPT framework [48]. Using NPT, we were able to identify where the intervention was addressing potential issues in implementation, for example by increasing patient willingness to self-monitor (coherence, individual specification); by training patients in the necessary skills required to undertake the work related to self-monitoring (collective action, skill set workability) and by ensuring patients felt confident in the reliability of the system (collective action, relational integration). As illustrated in the grey row of page 3 of Additional file 6, when considering patient adherence to medication titration, cognitive participation was coded as the most relevant determinant of change, with legitimation coded as the specific construct, as the evidence suggested it would be important to provide convincing evidence that medication adherence was the right thing to do. The secondary, deductive, theory-driven analysis is presented in Additional file 7; this did not identify any obvious requirements for further intervention content in the case of HOME BP (i.e. in addition to that identified through the person- and evidence-based planning activities).

#### Logic model

##### Purpose

To provide a diagram representing the hypothesised causal relationships mediating intervention outcomes [50].

##### Methods

The HOME BP logic model was constructed drawing upon the MRC process evaluation guidance [42]. Having specified clear HOME BP design objectives and key features using the guiding principles enabled us to be explicit about the assumptions which had guided the intervention development process, the problems to be addressed and the resultant intervention targets. Intervention components detailed in the behavioural analysis tables were summarised as intervention processes and incorporated into the logic model. To supplement the scoping and systematic review evidence from studies of digital interventions, additional non-systematic searches of the behavioural literature were conducted to identify the causal mechanisms relevant to our key behavioural targets (i.e. blood pressure self-monitoring, anti-hypertensive medication adherence and titration), in line with the recommendations made by Baxter and colleagues [44, 50].

A further 29 articles were identified, and the key findings arising from these studies were extracted and behavioural determinants coded against theories of behaviour change (see Additional file 8 for a three-page excerpt of literature relevant to key patient behaviours). This allowed us to select behaviour change theory specifically related to these determinants to guide the development of the logic model. The logic model visually represents the relationships between the intervention elements and theoretical constructs identified by the planning process and importantly conveys the complex inter-relationship between the patient and healthcare professional intervention components. The HOME BP logic model underwent several iterations incorporating team member and other stakeholder feedback.

##### Results

**Fig. 4:**
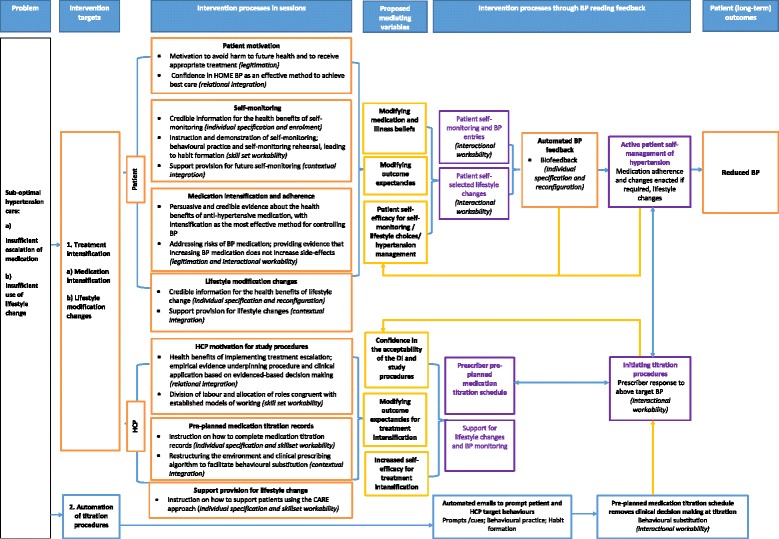
The HOME BP logic model. Note. The ‘Intervention processes in sessions’ section of the logic model condenses the information already presented in the behavioural analysis (available in Additional file 7). Within the logic model, these are organised around the patient and HCP target behaviours; summaries of the key BCTs used to promote each target behaviour are outlined in addition to the relevant NPT mechanism (presented in *brackets*)

The HOME BP logic model is presented in Fig. 4. To identify the likely causal mechanisms through which HOME BP would result in long-term behaviour change (and observable reductions in patient blood pressure), we identified both qualitative and quantitative literature examining the determinants of target behaviours (reported in Additional file 8) to develop an understanding of the potential mediating variables, drawing on additional psychological theory to map the proposed processes of change within the logic model.

Several key determinants were identified for blood pressure self-monitoring and anti-hypertensive medication adherence behaviours that mapped onto well-validated causal models. These comprised symptom perceptions, hypertension beliefs and treatment beliefs, which mapped onto the extended Common Sense Model (CSM) [51] and could also be theorised in terms of outcome expectancies in Social Cognitive Theory (SCT) [52]. In addition, there was evidence that self-efficacy (a central construct in SCT) is a key determinant of self-monitoring and medication adherence. We considered these two theories compatible and complementary, as the extended CSM provides more detail about the types of outcome expectancies likely to be relevant to illness management. Table 4 summarises the hypothesised relationships between symptom perceptions, illness beliefs and medication beliefs in hypertension.

**Table 4:** Summary of the relationships between hypertension symptoms, beliefs about hypertension and its treatment and potential mediating relationships identified within the literature searches to inform the logic model development

Symptom perception (illness identity)	Beliefs about hypertension	Beliefs about treatment/medication	Potential mediating relationship with self-management
No hypertension symptoms	• Less serious consequences, less concern, lower personal and treatment efficacy	• Treatment is not necessary • Medication signifies ill health	• Benefit of taking medication, or the health risk of non-adherence, may not be immediately noticeable in the short term • Objective BP readings may provide convincing evidence for the necessity of medication adherence • Medication acceptance is more likely when a clear benefit or rationale for anti-hypertensive medication is presented
Temporary increases in BP (fluctuating symptoms)	• High BP is perceived as separate to hypertension and occurring as a result of temporary modifiable factors such as stress or over-exertion	• Treatment only necessary when experiencing symptoms (i.e. to alleviate stress or to rest)	• BP monitoring would demonstrate that perceived fluctuations in symptoms are not a reliable indicator of when management is appropriate • Reductions in BP linked to appropriate self-management behaviours
Perception of hypertension-related symptoms (strong illness identity)	• High perceived consequences and emotional response to illness	• Poor medication adherence if a reduction in perceived symptoms are not observed in line with adherence behaviour	• Self-monitoring over extended periods (i.e. 7 days per week each month) may be important in decoupling perceived symptom experience from treatment beliefs • Feeling better and the perceived benefits of anti-hypertensive medication were identified as reasons to take anti-hypertensive medication

## Discussion

This paper has described how we combined evidence-, theory- and person-based approaches to the development of a complex intervention to support patient self-management of hypertension. The importance of basing interventions on existing evidence—synthesised by means of systematic reviews—has been acknowledged for some time [53]. It is now also widely recognised that for successful complex intervention development, it is vital to engage in theoretical modelling, in order to identify and change the determinants of behaviour [26]. In addition, there is now growing awareness of the need for a Person-Based Approach, which fosters a detailed, in-depth understanding of the perspective of the people who will use the intervention [27]. The Person-Based Approach is a particular approach to user-centred design that is intended specifically for development of complex behavioural interventions, and therefore focuses principally on user perspectives on the intended behaviour change and its context. It is therefore particularly suitable for combining with intervention development approaches that draw on behavioural theory. This paper has illustrated the value of integrating insights from these three approaches (theory-, evidence- and person-based) in the development of HOME BP.

Each of the six elements of our integrated approach (see Fig. 1) contributed valuable and complementary insights, whilst bringing together these insights increased the confidence and clarity of our decision-making. Two aspects of this process were particularly useful. First, synthesising qualitative and quantitative evidence from our reviews and primary research enabled us to ground all elements of our behavioural analysis and selection of behaviour change techniques in a context-specific appreciation of what barriers and facilitators for self-management using digital health interventions were especially relevant to this intervention. Second, our multi-layered theoretical modelling enabled us to articulate three different but interlinked perspectives on our intervention. Our guiding principles succinctly summarised the distinctive design objectives and features of HOME BP. Our behavioural analysis provided a complete, systematic documentation of the determinants of behaviour and how these were addressed by the behaviour change techniques included in the intervention. Finally, our logic model presented an overview of the intervention, showing the linkage between patient and health professional behaviour and demonstrating how the behaviours and their determinants mapped onto both psychological and sociological theoretical frameworks.

### Future applications

Although each of the elements of our integrated theory-, evidence- and person-based approach to intervention planning can make an important contribution, the most appropriate way to undertake each element is likely to differ widely depending on the particular context of each intervention. Although we have taken all of the steps outlined above in the planning and development of HOME BP, it may or may not be necessary to undertake primary qualitative or mixed methods research at the start, depending on the quality and relevance of the existing evidence base—although it will always be important to use primary mixed methods research to evaluate and refine intervention elements initially developed on the basis of evidence and theory [27, 34]. Methodological advances will also influence how this integrated approach can be applied in future; for example, new methods of systematic review and evidence synthesis are being developed to assist the identification of effective intervention ingredients and important contextual factors in successful intervention implementation [50]. Time and resource constraints will inevitably influence how the intervention planning process can be carried out, and when these are limited, it will only be possible to engage in rapid, ‘light touch’ evidence collation and theoretical modelling. However, in this integrated, iterative approach to intervention planning, the order in which the elements are undertaken can be flexibly adapted as required; for example, if there is insufficient time to complete intervention planning before intervention development must commence (as in the case of HOME BP), then preliminary theoretical modelling can be based on partial evidence, and then updated and refined once evidence collation and analysis is complete.

## Conclusion

Our integrated approach to intervention development, combining theory-, evidence- and person-based approaches, increased the clarity, comprehensiveness and confidence of our theoretical modelling and enabled us to ground our intervention in an in-depth understanding of the barriers and facilitators most relevant to this specific intervention and user population.

## Additional files

Additional file 1: Full methods for work stream 1: collating and analysing evidence. (DOCX 33 kb)
Additional file 2: Scoping search of qualitative literature for digital intervention use in long-term health conditions (such as hypertension, asthma, diabetes and associated conditions) and final article inclusion. (DOCX 21 kb)
Additional file 3: Excerpt from the rapid review of qualitative studies of digital interventions for long-term condition self-management. (DOCX 21 kb)
Additional file 4: Synthesis of the qualitative literature to identify potential barriers and facilitators for key target behaviours, acceptability and engagement for digital self-management interventions. (DOCX 25 kb)
Additional file 5: Excerpts from the quantitative review of evidence for intervention components used for hypertension self-management. (DOCX 18 kb)
Additional file 6: Behavioural analysis of HOME BP using the Behaviour Change Wheel (BCW) and Normalisation Process Theory (NPT). (DOCX 26 kb)
Additional file 7 Applying the NPT and BCW theoretical frameworks to the HOME BP intervention content: an analysis of patient intervention components for each NPT and BCW construct. (DOCX 19 kb)
Additional file 8: Excerpts from the key findings arising from qualitative and quantitative literature searches to identify studies examining the potential determinants of key HOME BP patient behaviours. (DOCX 17 kb)
