# Supplementary material for: Intervention planning for a digital intervention for self-management of hypertension: a theory-, evidence- and person-based approach
Source: Implement Sci. 2017 Feb 23;12:25. doi: 10.1186/s13012-017-0553-4 (PMC5324312; doi:10.1186/s13012-017-0553-4)
Supplement: Additional file 1: — Full methods for work stream 1: collating and analysing evidence. (DOCX 33 kb) [file 13012_2017_553_MOESM1_ESM.docx]

**Additional file 1: Full methods for Workstream 1: Collating and analysing evidence**

*1.1 Primary mixed methods research*

*1.1.1 Methods*

A small feasibility study was carried out before planning for HOME BP commenced, using online materials for patients (blood pressure self-monitoring instructions and titration feedback) that closely corresponded to the written materials used in TASMINH2 [1]. These online materials were supplemented by an internet-delivered healthy behaviour change programme supporting weight management and physical activity; this was adapted from the POWeR weight management intervention (which is described in detail elsewhere [2]) with the addition of brief modules on salt and alcohol reduction. Patients were encouraged to set weekly goals for behaviour change, and received personalised feedback based on their reported goal-related progress. The intervention was trialled on 50 participants from 8 practices, and qualitative interviews were carried out with 16 patients (aged 46-83; n= 9 male) and 3 healthcare professionals (n=3 female). Debriefing focus groups (not recorded) were also carried out at practice meetings with a further 8 health professionals. Open-ended questions elicited views of the intervention, focusing particularly on issues of acceptability and feasibility [3].

*1.1.2 Outputs*

Key issues arising from the feasibility study are summarised in Table 1, which also explains how HOME BP was designed to address these issues. A crucial insight from this stage of the intervention planning was that translating the TASMINH2 intervention into an effective internet-delivered intervention was not simply a matter of transferring written materials online. It proved difficult for primary care staff to implement the intervention independently, without any input from the research team. To encourage primary care staff and patients to adhere to the titration protocol it was necessary to put in place easily implemented online procedures, supported by safety checks and reassurance (e.g. about side-effects and medical supervision), to ensure that both patients and medication prescribers would feel motivated and confident to undertake titration without a consultation.

*1.2 Qualitative synthesis*

*1.2.1 Methods*

In the early stages of intervention planning we immediately commenced a scoping review to identify qualitative studies examining patient, healthcare professional and other stakeholder perspectives and experiences of using tele-medicine or digital interventions to support self-management in long-term health conditions. The review focused originally on cardiovascular and respiratory conditions, as these were most relevant to the interventions for hypertension and asthma within the research programme which funded the development and evaluation of HOME BP. To broaden our evidence base, we then decided to include other conditions requiring similar behaviours for successful self-management (such as self-monitoring, medication adherence and healthy behaviour changes); patient experiences of digital interventions for chronic heart failure, heart disease, COPD, diabetes and chronic low back pain were therefore also represented.

The search initially identified 1287 articles which were reduced to 19 full-text papers following title and abstract screening. Full details of the electronic databases searched, search terms used and the full list of articles included in the synthesis are provided in Appendix 2. An initial rapid review of the literature was necessary to ensure that the evidence identified could be quickly incorporated in to the initial intervention planning and development phases [4]. Data extraction included a description of the intervention components (where available), evidence of facilitators, evidence of barriers, and other findings reported within the paper (see Appendix 3 for a 4 page excerpt from the extensive data extraction table). Thematic analysis was conducted on the extracted data, and all coding agreed with a second author (KM) (presented in Appendix 4). A full qualitative review and meta-synthesis of the literature was later conducted in line with the project time constraints [5].

*1.2.2 Outputs*

Five key themes emerged from the initial qualitative synthesis, and findings were organised around facilitators and barriers relating to each theme. Additional information regarding how facilitators were (or could be) utilised, and the ways in which barriers were (or could be) addressed was also recorded. The full table outlining barriers and facilitators relating to both patient and healthcare professional engagement with digital interventions (or equivalent systems) for patient self-management is provided in Appendix 4. Selected examples of how this evidence informed intervention planning are provided below.

The evidence suggested that healthcare professional confidence in the system was an important factor to consider, particularly with reference to the reliability and accuracy of readings. We therefore emphasised in the health professional training materials that home blood pressure readings were more accurate than clinic readings as the basis for clinical decision making and that the titration procedures were based on current gold standard procedures for hypertension control [1]. We also used the qualitative evidence to provide further support for some HOME BP design decisions suggested by our primary qualitative research. For example, the qualitative literature confirmed that the information provided in HOME BP would need to be motivating, providing strong evidence for the benefit of titrating medications, and addressing potential concerns about unwanted side effects.

- 1. *Quantitative systematic review*

*1.3.1 Methods*

A quantitative systematic review was conducted to identify digital interventions whose primary or secondary outcomes included reduction in blood pressure [6]. For the purposes of the intervention planning and development process, relevant papers that were excluded against review criteria (for example, non-interactive telemedicine interventions) were also used to inform HOME BP planning. Following a similar approach to that reported by Baxter and colleagues [7], we also considered non-trial sources such as systematic reviews and meta-analyses identified by the search and the research team, and extracted information from further relevant papers identified by the research team throughout the development phase of HOME BP.

Detailed information about the intervention components and study procedures was extracted (where relevant) and tabulated for the following categories: reported self-monitoring schedules, methods of blood pressure feedback, titration procedures, type and level of behavioural support, and the length of the intervention (study follow-up). Descriptions of the control group were also extracted where possible. Reported efficacy in terms of reductions in systolic blood pressure and diastolic blood pressure was recorded, in addition to any further findings (such as cost-effectiveness calculations). Selected excerpts from these extensive extraction tables are provided in Appendix 5. Evidence identified from all sources was incorporated into the intervention planning tables when relevant to intervention component design features, and cross-referenced in the record of decision making. Specific examples of this will be provided in the results section below.

*1.3.2* *Outputs*

The review confirmed that self-management interventions can lead to reductions in blood pressure [6]; evidence arising from the quantitative literature searches was also fed immediately into the development of HOME BP. For example, a key issue arising from the quantitative literature was that previous interventions reporting efficacious reductions in participant blood pressure had utilised relatively intensive behavioural support [1, 8-10], with some providing behavioural support as frequently as every two weeks until BP was controlled [11, 12]. Whilst the evidence suggested that support was a beneficial addition to self-monitoring for improved patient outcomes, recent meta-analyses suggested it was unclear what the optimum level of support is [13]. Moreover, the planning and development of HOME BP had to balance the potential benefits of health professional support with what would be feasible and cost-effective to offer within a UK primary care context. Input was welcomed from all stakeholders (which included primary care clinicians, experts in behavioural science, patient representatives, and experts in hypertension) regarding the essential level of support required to increase adherence without increasing face-to-face consultation, considering the feasibility requirements for potential future NHS implementation. As a result, it was decided that face-to-face support would be offered for the first week of self-monitoring and after the introduction of behaviour changes, as these are key times within the intervention when patients are likely to require additional support. It was decided that regular support (every 4 weeks) would be provided to the patient by email, and that the patient would be able to request additional support at any time through the HOME BP programme, restricted to a maximum of six face-to-face support sessions.

References

1. McManus, R.J., et al., *Telemonitoring and self-management in the control of hypertension (TASMINH2): a randomised controlled trial.* The Lancet, 2010. **376**(9736): p. 163-172.

2. Lloyd, S., et al., *Losing weight online with POWeR: a randomised controlled trial of a web-based behavioural intervention in a community setting.* The Lancet, 2013. **382, Supplement 3**: p. S62.

3. Moore, G., et al., *Process evaluation of complex interventions*, U.M.R.C.M. guidance, Editor. 2014

4. Ganann, R., D. Ciliska, and H. Thomas, *Expediting systematic reviews: methods and implications of rapid reviews.* Implement Sci, 2010. **5**: p. 56.

5. Morton, K., et al., *Using digital interventions for self-management of chronic physical health conditions: A meta-ethnography review of published studies.* Patient Education and Counseling, 2016.

6. McLean, G., et al., *Digital interventions to promote self-management in adults with hypertension systematic review and meta-analysis.* Journal of Hypertension 2016 **34**(4): p. 600-612.

7. Baxter, S.K., et al., *Synthesising diverse evidence: the use of primary qualitative data analysis methods and logic models in public health reviews.* Public Health, 2010. **124**(2): p. 99-106.

8. Bosworth, H.B., M.K. Olsen, and G.J.M.e. al., *Two Self-management Interventions to Improve Hypertension Control: A Randomized Trial.* Annals of Internal Medicine 2009. **151**: p. 687-695.

9. Bennett, G.G., et al., *Web-based weight loss in primary care: a randomized controlled trial.* Obesity (Silver Spring), 2010. **18**(2): p. 308-13.

10. Bennett, G.G., et al., *Obesity treatment for socioeconomically disadvantaged patients in primary care practice.* Arch Intern Med, 2012. **172**(7): p. 565-74.

11. Green, B.B., et al., *Effectiveness of home blood pressure monitoring, Web communication, and pharmacist care on hypertension control: a randomized controlled trial.* Jama, 2008. **299**(24): p. 2857-67.

12. Margolis, K.L., et al., *Effect of Home Blood Pressure Telemonitoring and Pharmacist Management on Blood Pressure Control. A Cluster Randomized Clinical Trial.* JAMA, 2013. **310**(1): p. 46-56.

13. Uhlig, K., et al., *Self-Measured Blood Pressure Monitoring in the Management of Hypertension. A Systematic Review and Meta-analysis.* Annals of Internal Medicine, 2013 **159**(3): p. 185 - 194.
