# Supplementary material for: Intervention planning for a digital intervention for self-management of hypertension: a theory-, evidence- and person-based approach
Source: Implement Sci. 2017 Feb 23;12:25. doi: 10.1186/s13012-017-0553-4 (PMC5324312; doi:10.1186/s13012-017-0553-4)
Supplement: Additional file 2: — Scoping search of qualitative literature for digital intervention use in long-term health conditions (such as hypertension, asthma, diabetes and associated conditions) and final article inclusion. (DOCX 21 kb) [file 13012_2017_553_MOESM2_ESM.docx]

**Additional file 2: Scoping search of qualitative literature for digital intervention use in long-term health conditions (such as hypertension, asthma, diabetes and associated conditions) and final article inclusion**

| **Database** | **E-health terms** | **Intervention terms** | **Qualitative methods terms** | **Chronic illness terms** | **Records identified** | **Records Excluded** | **n included** |
| --- | --- | --- | --- | --- | --- | --- | --- |
| Web of Science | Internet  Online  Digital  Web  E-health  Telemonit*  Computer*  Technolog*  Telecommunication*  Multimedia  PC  Website  WWW  Cellular Phone  Cell phone  Mobile  Smartphone  Smart phone  Electronic  Ehealth  Mhealth  M-health  Telemedicine  Text messag*  Email  Telehealth  Teletherap* | Intervention  Programme  Self-management  Self management  health education  Self care  Self-care  Self-monit*  Self monit* | Qualitative  Interview  Focus group  Experience  View  Perspective  Feedback  Ethnograph*  Thematic  Theme  Mixed methods  Usability  Thinkaloud  Think aloud  Open-ended  Semi-structured | Asthma  Hypertens*  Blood Pressure | 342 | 336 | 6 |
| Medline, Psychinfo and CINAHL (keyword search) | (as above) | (as above) | (as above) | (as above) | 523 | 518 | 5 |
| PsychINFO (thesaurus search) | Internet, communication systems, information systems,  computer applications, computer mediated communication, online therapy, telecommunications media,  telemedicine, websites,  computer assisted therapy,  computer mediated communication, computer programming | Self -care skills,  self management, self monitoring |  | Asthma, hypertension, essential hypertension, blood pressure, diastolic pressure, systolic pressure, chronic illness | 26 | 26 | 0 |
| Embase (thesaurus search) | Internet,  online system, computer program, computer,  computer assisted drug therapy, computer system, computer assisted therapy | Self care,  self help,  self medication |  | Asthma, hypertension, elevated blood pressure, chronic disease | 149 | 147 | 2 |
| CINHAL (thesaurus search) | Online systems, online services, internet,  world wide web, computer systems, therapy computer assisted | Self care |  | Asthma, Hypertension (exploded), chronic disease | 148 | 147 | 1 |
| Medline (thesaurus search) | Internet,  online systems, computers, computer systems | Self-care |  | Asthma, Hypertension, Blood pressure, Chronic disease | 125 | 124 | 1 |
| Reference lists of identified papers | n/a | Telemonit* | n/a | n/a | 4 | 0 | 4 |

**Final articles included within the synthesis:**

| **Author (year)** | **Title** | **Journal reference** | **Source** |
| --- | --- | --- | --- |
| Ahern, Stinson, Uebelacker, Wroblewski, McMurray & Eaton (2012) | E-Health Blood pressure control program | The Journal of Medical Practice Management; 28, 91-100. | Keyword Search |
| Anhøj & Nielsen (2004) | Quantitative and Qualitative Usage Data of an Internet-Based Asthma Monitoring Tool | J Med Internet Res;6(3):e23.  doi:10.2196/jmir.6.3.e23 | Keyword search |
| Armstrong, Hearnshaw, Powell & Dale (2007) | Stakeholder Perspectives on the Development of a Virtual Clinic for Diabetes Care: Qualitative Study | J Med Internet Res;9(3):e23.  doi:10.2196/jmir.9.3.e23 | Embase thesaurus |
| Bostock, Hanley, McGown, Pinnock, Padfiels & McKinstry (2009) | The acceptability to patients and  professionals of remote blood pressure  monitoring using mobile phones | Primary Health Care Research & Development 2009; 10: 299–308.  doi:10.1017/S1463423609990107 | Reference list searching |
| Cassimatis, Kavanagh & Smith (2014) | Perceived Needs for Supported Self-management of Type 2 Diabetes: A Qualitative Investigation of the Potential for a Web-based Intervention | Australian Psychologist 49 (2014) 75–85. doi:10.1111/ap.12050 | Web of science |
| Fairbrother, Ure, Hanley, McCloughan, Denvir, Sheikh & McKinstry (2014) | Telemonitoring for chronic heart failure: the views of patients and healthcare professionals – a qualitative study | Journal of Clinical Nursing,  doi: 10.1111/jocn.12137 | Reference list searching |
| Grant, Pandiscio, Pajolek, Woulfe, Pelletier,Kvedar, &  Park (2012) | Implementation of a web-based tool for patient medication self-management: the Medication Self-titration Evaluation  Programme (Med-STEP) for blood pressure control | Inform Prim Care; 20(1): 57–67. | Web of science |
| Halifax, Cafazzo, Irvine, Hamill, Rizo, McIsaac, Rossos & Logan (2007) | Telemanagement of hypertension: A qualitative assessment of patient and physician preferences | Can J Cardiol; 23(7): 591–594. | Embase thesaurus |
| Hanley, Ure, Pagliari, Sheikh & McKinstry (2013) | Experiences of patients and  professionals participating in the HITS  home blood pressure telemonitoring  trial: a qualitative study | BMJ Open; 3: e002671. doi:10.1136/bmjopen-2013-002671 | Keyword search |
| Hartmann, Sciamanna, Blanch, Mui, Lawless, Manocchia, Rosen & Pietropaoli (2007) | A Website to Improve Asthma Care by Suggesting Patient Questions for Physicians: Qualitative Analysis of User Experiences | J Med Internet Res ;9(1):e3  doi:10.2196/jmir.9.1.e3 | Keyword search |
| Jones, Greenfield, Bray, Baral-Grant, Hobbs,  Holder, Little, Mant, Virdee, Williams & McManus | Patients’ experiences of self-monitoring blood pressure and self-titration of medication: the TASMINH2 trial qualitative study | British Journal of General Practice 62(595):e135-42. DOI: 10.3399/bjgp12X625201. | Keyword search |
| Kerr, Murray, Noble, Morris, Bottomley, Stevenson, Patterson, Peacock, Turner, Jackson, & Nazareth (2010) | The Potential of Web-based Interventions for Heart Disease Self-Management: A Mixed Methods Investigation | J Med Internet Res;12(4):e56  doi:10.2196/jmir.1438 | Web of science |
| Langstrup (2008) | Making connections through online asthma monitoring | Chronic Illness; 4; 118-126.  DOI: 10.1177/1742395308092480 | Keyword search |
| Pinnock, Slack, Pagliari, Price & Sheikhz (2007) | Understanding the potential role of mobile phone-based monitoring on  asthma self-management: qualitative study | Clinical and Experimental Allergy, 37(5):794-802. | Reference list searching |
| Rahimpour, Lovell, Celler & McCormick (2008) | Patients’ perceptions of a home telecare system | International Journal of Medical Informatics; 77; 486 -498.  doi:10.1016/j.ijmedinf.2007.10.006 | Web of science |
| Ure, Pinnock, Hanley, Kidd, McCall, Smith, Tarling, Pagliari, Sheikh, MacNee & McKinstry (2011) | Piloting tele-monitoring in COPD: a mixed methods exploration of issues in design and implementation | Primary Care Respiratory Journal; 21(1):57-64. doi: 10.4104/pcrj.2011.00065. | Reference list searching |
| Urowitz, Wiljer, Dupak, Kuehner, Leonard, Lovrics, Picton, Seto & Cafazzo (2012) | Improving Diabetes Management With a Patient Portal: Qualitative Study of a Diabetes Self-Management Portal | J Med Internet Res ;14(6):e158  doi:10.2196/jmir.2265 | Web of science |
| Voncken-Brewster, Tange, Moser, Nagykaldi, Vries &  van der Weijden (2014) | Integrating a tailored e-health self-management application for chronic obstructive pulmonary disease patients into primary care: a pilot study | BMC Family Practice, 15:4  http://www.biomedcentral.com/1471-2296/15/4 | Web of science |
| Zuffery & Schulz (2009) | Self-management of chronic low back pain: An exploration of the impact of a patient-centered website | Patient Education and Counseling 77; 27–32. doi:10.1016/j.pec.2009.01.016 | CINAHL thesaurus |
