# Supplementary material for: Intervention planning for a digital intervention for self-management of hypertension: a theory-, evidence- and person-based approach
Source: Implement Sci. 2017 Feb 23;12:25. doi: 10.1186/s13012-017-0553-4 (PMC5324312; doi:10.1186/s13012-017-0553-4)
Supplement: Additional file 3: — Excerpt from the rapid review of qualitative studies of digital interventions for long-term condition self-management. (DOCX 21 kb) [file 13012_2017_553_MOESM3_ESM.docx]

**Additional file 3: Excerpt from the rapid review of qualitative studies of digital interventions for long-term condition self-management**

| **Study** | **Intervention description** | **Evidence of barriers**  *Barriers* (methods to address barriers provided in corresponding brackets) | **Evidence of facilitators**  *Facilitators* (ways in which these could be incorporated in corresponding brackets) | **Other findings reported in the paper** |
| --- | --- | --- | --- | --- |
| Ahern et al (2012) – *hypertension* | Patients given a BP monitor and asked to monitor BP twice a day for 7 days.  2 digital intervention conditions:  1. Access to personal health record online and web-portal for BP monitoring  2. As above plus additional support from a patient navigator (for recording BP, taking medication as prescribed and attending visits with the HCP). Support was provided monthly and/or event-driven (i.e. as a result of BP or goal status). | Themes arising from patient focus groups (for tailoring the web-based portal and functionality):   - *Lack of understanding of the importance and frequency of BP monitoring* - *Misperceptions about the need for ongoing medication usage* (patient education videos and resource material) | Themes arising from patient focus groups (for tailoring the web-based portal and functionality):   - *Need for simple and clear BP readings and their interpretation* (website designed to graph BP and use of analytical tools, and traffic light system for BP readings and flashing light for 2 consecutive readings above 180/120mmHg) - *Tools to give HCP access to BP readings* (HCP interface with access to BP readings) - *Perceived value of support from HCP team* (messaging system to send text/ email prompts from HCP staff; communication templates created; monthly face-to-face contact with patient navigator) | Piloted 28 patients:   - DI with support appeared to be effective at overcoming clinical inertia (speculated this was as a result of optimising titration and feedback regarding BP trajectory). - Providing HCPs with information summaries enhances communication between clinicians and patients (Ahern et al, 2011) |
| Grant et al (2012) – *hypertension/ diabetes* | Med-STEP intervention consisted of a patient web-based interface that graphically represented BP readings, an automated upload system, and a HCP interface (with access to patient results and enabled secure patient-HCP messaging). Also included a medication treatment algorithm (and associated info such as BP thresholds) which patients could view – known as ‘my treatment pathway’. | Patient concerns about using DI:   - *Detrimental effect on doctor-patient relationship* (regular HCP appointments; medication treatment pathway designed by own/ regular HCP) - *Variability in blood pressure readings* (home monitor readings used to inform treatment decisions based on average of several readings; automated BP reading cuffs) - *Correctly following treatment pathways* (automated BP uploads, pathway available on website, algorithm run by computer, verified by physician PI) - *Medication side effects* (all medication chosen by own/ regular HCP; possible side effects listed in treatment pathway; instructed to call practice if experiencing side effects; highlighting no increased risk of side effects relative to usual care) - *Too much effort/ don’t want responsibility* (selective programme; automated home monitoring device and pathway; regular HCP appointments – DI does not replace usual visits) | Themes arising from patient focus groups (relating to the benefits of medication titration process):   - *Awareness* – knowing the sequence of medication steps in titration process was appealing for learning about the illness and reducing anxiety - *Engagement* – DI increasing patient sense of responsibility for BP management - *Motivation* – DI increasing motivation leading to better disease control - *Convenience* – DI avoids delay in titration between appointments | Piloted Med-STEP with 16 participants:   - 3 participants who had medication increases had mean SBP/DBP reduction of 8.7/5.7mmHg - Patients thought the intervention was useful and would recommend to others - Frustration with technical issues - Patients reluctant to make medication changes |
| Halifax et al (2007) – *hypertension/ diabetes* | Participants were asked to provide opinions based upon a prototype hypertension tele-management system. | Themes arising from patient focus groups:   - *Concerns about medication* – patients expressed concerns about being over-medicated - *Lack of education* - patients recognised lack of working knowledge about hypertension, related terms, and health consequences of high or low BP and acknowledged this compromised their ability to address concerns   Themes arising from physician focus groups:   - *Accuracy of home BP readings* – physicians were concerned about differences in home and clinic readings, viewing clinic readings as more reliable - *Patient acceptance* – concerns that patients with lower BP home readings were more likely to resist taking additional medications when clinic readings were high - *Patient anxiety* – concerns that some patients would over focus on BP readings and exacerbate condition. - *Impact on workload* – Concern system would increase workload, did not want to disrupt current workflow and systems. Increasing clinic appointments was undesirable - *Patient access to information* - some HCPs did not want patients to access as much information as them as this may trigger non-urgent appointments | Themes arising from patient focus groups:   - *Reliability and accuracy* – (patients liked that the system readings were reliable and accurate, that using the system may show a better picture of BP readings than clinic ‘snapshots’) - *Access to BP readings* – (patients liked idea that could download and print readings) - *Education* – (more education would increase ability to make more responsible health choices and collaboration with HCPs).   Themes arising from physician focus groups:   - *Reliability* – (physicians liked the objectivity of the system, that readings would be valid) - *Facilitating communication* – (physicians liked the opportunity to provide immediate feedback) - *Format of BP readings* – (preferred if information on BP readings could be provided in numerous forms – graphic, tabular, numerical and text) | - Over half of patients (n =24 hypertension and type 2 diabetes, aged 30-85) had a home computer and all had sent an email - Not all had mobile phones and most only used basic functions - Many patients monitored BP at home and had used/ purchased a home BP monitoring device and were willing to engage in frequent monitoring |
| Jones et al (2012) – *hypertension* | TASMINH2 – home blood pressure self-monitoring and self-titration of anti-hypertensive drugs according to a pre-planned titration schedule. | - *Concerns about medication* – reluctance to increase medication was due to side effects and being aware they would take increased doses for the rest of their lives - *Titrating in borderline cases* – Frustration when reading were ‘just’ amber and this would trigger a titration – sometimes would not titrate in borderline cases but think carefully about it instead. - *Patient confidence* - lack of confidence in titrating, choosing to re-consult before doing so. | - *Understanding and knowledge* – TASMINH2 trial helped patients to understand BP and its management. Patients learnt about BP variability – and became unconvinced of appropriateness of making med changes based on one/ a few BP readings - *Patient confidence in self-management* – Patients became more confident at measuring and interpreting BP readings.   – Patients felt greater control at self-titrating – but only as a result of knowing this was pre-determined by their doctor  – Trial procedures gave patients the confidence to know when to ask for advice  – Patients felt home monitoring was more natural, relaxed, carefully taken and controlled. | - Most people knew of the risks but had felt that they were not at risk because their BP was treated (although not controlled) or because of a lack of symptoms |

BP = blood pressure; DBP = diastolic blood pressure; DI = digital intervention; HCP = Healthcare professional; SBP = systolic blood pressure
