# Supplementary material for: Intervention planning for a digital intervention for self-management of hypertension: a theory-, evidence- and person-based approach
Source: Implement Sci. 2017 Feb 23;12:25. doi: 10.1186/s13012-017-0553-4 (PMC5324312; doi:10.1186/s13012-017-0553-4)
Supplement: Additional file 4: — Synthesis of the qualitative literature to identify potential barriers and facilitators for key target behaviours, acceptability and engagement for digital self-management interventions. (DOCX 25 kb) [file 13012_2017_553_MOESM4_ESM.docx]

**Additional file 4: Synthesis of the qualitative literature to identify potential barriers and facilitators for key target behaviours, acceptability and engagement for digital self-management interventions**

**Facilitators**

| **Facilitator** | Qualitative information on likely facilitators  How facilitators were/ could be utilised |
| --- | --- |
| **Key behaviour:** BP Self-monitoring | |
| *Simple, clear BP reading feedback*  (Ahern et al, 2012; Bostock et al, 2010 ; Halifax et al, 2007) | - Website designed to graph BP and use of analytical tools, - Traffic light system for BP readings - Flashing light for 2 consecutive readings above 180/120mmHg - Explaining the meaning of readings in clear language - Outlining that high readings do not mean immediate risk - Download and print BP readings |
| *Appropriate training*  (Grant et al, 2012;Rahimpour et al, 2008 ; Hanley et al, 2013; Jones et al, 2012) | - Patients report training as important for self-monitoring |
| *Increased concern*  (Hanley et al, 2013) | - Anxiety about BP and concern about hypertension increased monitoring |
| *Help patients understand BP/ management*  (Bostock et al, 2010; Jones et al, 2012) | - HBPM helps patients to make lifestyle and medication changes - Self-monitoring and self-titration helped to understand BP and its management |
| *Reliability and accuracy*  (Halifax et al, 2007; Hanley et al, 2013; Jones et al, 2012) | - Patients liked that readings were reliable and accurate - Home monitoring more natural and relaxed - Patients learnt about BP variability - More accurate picture of BP than clinic ‘snapshot’ (carefully taken and controlled) - Patients and HCPs felt that decisions based upon multiple readings were likely to be more accurate (more appropriate basis for action) |
| *Free-text responses important*  (Langstrup, 2008 – asthma) | - Allowed patient to add in additional information for the HCP- to bridge the gap between everyday life and the clinic |
| *Speed up intensification*  (Bostock et al, 2010; Hanley et al, 2013) | - Home monitoring and feedback can facilitate rapid medication tailoring - Home monitoring useful in new or borderline cases - Persuasive evidence for action |
| **Key behaviour:** Self-management (more generally) | |
| *Knowledge –*  (Halifax et al, 2007; Cassimatis, Kavanagh & Smith, 2014 - diabetes) | - Patients felt more education would increase ability to make more responsible health choices and collaboration with HCPs - Access to a broad range of informational support facilitates self-management |
| *Knowing health status over time*  (Fairbrother et al, 2013 – chronic heart failure; Rahimpour et al (2008) | - Led to feeling better informed and more knowledgeable - Patient reports of reassurance about health status |
| *Developing a routine* (Cassimatis, Kavanagh & Smith, 2014 - diabetes) | - Having a plan and following a routine was beneficial (monitoring, medication adherence, physical activities) |
| *Patients actively involved in own care*  (Haartman et al, 2007 – asthma) | - Patients more aware of treatment opportunities and the need for changes in the frequency or style of their involvement in their condition |
| *Motivation*  (Grant et al, 2012; Hanley et al, 2013) | - Patients expected that a self-management programme would increase motivation to control health condition. - Participation increased motivation for general lifestyle change - Home monitoring led to increased motivation for self-care |
| *Increased self-confidence*  (Jones et al, 2012; Haartman et al, 2007 – asthma) | - Patients become more confident at monitoring and interpreting BP readings. - Patients felt more confident in understanding asthma and its treatment |
| *Patient characteristics*  (Anhas & Neilsen, 2004 – asthma) | - ‘Controllers’ want to get control of their illness, establish daily routines, control and monitor to reduce worry. |
| **Key behaviour:** Medication adherence and intensification | |
| *Awareness of titration schedule* –  (Grant et al, 2012; Jones et al, 2012) | - Knowing the sequence of medication steps in titration process was appealing for learning about the illness and reducing anxiety - Patients felt greater control at self-titrating – but only as a result of knowing this was pre-determined by their doctor |
| **Engagement:** Patient and health professional experiences of DI (or other equivalent system) | |
| *Increasing patient sense of responsibility for BP* management (Grant et al, 2012; Bostock et al, 2010) | - **Emphasising shared responsibility between GP and patient** |
| *Perceived value of support from HCP team*  (Haartman et al, 2007 – asthma; Fairbrother et al, 2013 – chronic heart failure) | - Reassured by the surveillance and support from the practitioner - Messaging system to send text/ email prompts from HCP staff - Communication templates created - Monthly face-to-face contact with patient navigator |
| *Improve healthcare access*  (Bostock et al, 2010; Rahimpour et al, 2008 ; Fairbrother et al, 2013 – chronic heart failure; Ahern et al, 2012; Langstrup, 2008 – asthma) | - Reduce no of routine visits needed in practice - Early intervention if necessary - Cost savings - HCP – felt facilitated more pro-active clinical management - Programmes give HCP access to patient data (link up the HCP and patient) |
| *Facilitate interactions between patient and HCPs*  (Hanley et al, 2013; Jones et al, 2012; Haartman et al, 2007 – asthma; (Anhas & Neilsen, 2004 – asthma; Kerr et al, 20102 – heart disease) | - Improved communication with the GP - Patient confidence to know when to ask for advice - Patients engaged in interactions more and had more positive perception of interactions - Facilitate GP conversation around raised BP if evidence of continued BP above target. - Email contact worked well - GP transitioned from expert to consultant (used to identify problems and cures) - Sometimes online interaction preferable for some patients |
| *Convenience of DI -*  (Grant et al, 2012; Rahimpour et al 2008) | - Avoids delay in titration between appointments - Less travel time |
|  |  |
| **Acceptability:** Health professional confidence in the online system | |
| *Objectivity of system readings*  (Halifax et al, 2007) | - Liked information provided in various formats (i.e. graphs, tables, number and text) |
| *Opportunity to provide quicker feedback on BP readings*  (Halifax et al, 2007) |  |

**Barriers**

| **Barrier** | Qualitative information on likely barriers  How barriers were addressed |
| --- | --- |
| **Key behaviour:** BP Self-monitoring | |
| *Feedback messages*  (Anhoj & Neilsen, 2004 – asthma; Hanley et al, 2013) | - Automated messages were not seen as valuable - Wording of feedback messages is important - patients preferred content focussed on health status (rather than giving advice) |
| *Potential increase in anxiety*  (Bostock et al, 2010; Halifax et al, 2007; Fairbrother et al, 2013 – chronic heart failure) | - Particularly if warning messages received after high BP readings - GP concerns that some patients would over focus on BP readings and exacerbate condition |
| *Lack of understanding of the importance and frequency of BP monitoring*  (Ahern et al, 2012) | - Patient education videos and resource material |
| *Concern about variability in blood pressure readings*  (Grant et al, 2012;Rahimpour et al, 2008) | - Reliability of home monitoring equipment - Home monitor readings used to inform treatment decisions based on average of several readings - automated BP reading cuffs |
| *Well-controlled BP*  (Bostock et al, 2010) | - less likely to see the benefits of self-monitoring |
| **Key behaviour:** Self-management (more generally) | |
| *Lack of working knowledge about hypertension, related terms, and health consequences of high or low BP*  (Halifax et al, 2007; Cassimatis, Kavanagh & Smith, 2014 - diabetes) | - Increased emotional difficulties to coping |
| *Information about disease progression*  *(*Cassimatis, Kavanagh & Smith, 2014 - diabetes) |  |
| *Self-management when asymptomatic*  (Anhoj & Neilsen, 2004 – asthma) | - Patients found it difficult to use medication on a daily basis (i.e. when they are not experiencing symptoms). |
| *Lack of motivation*  (Langstrup, 2008 – asthma) | - GPs blamed a lack of patient motivation and interest for poor implementation in the practice |
| *Patient characteristics*  (Anhas & Neilsen, 2004 – asthma) | - ‘Neglecters’ – don't want to think about the disease and feel better by not focussing on it – think this is better for their health. |
| **Key behaviour:** Medication adherence and intensification | |
| *Medication side effects*  (Grant et al, 2012; Jones et al, 2012) | - all medication chosen by own/ regular HCP - possible side effects listed in treatment pathway - instructed to call practice if experiencing side effects - No increased risk of side effects relative to usual care |
| *Misperceptions about the need for ongoing medication usage*  (Ahern et al, 2012; Jones et al, 2012) | - Most people knew of the risks but had felt that they were no at risk because their BP was treated (although not controlled) or because of a lack of symptoms - patient education videos and resource material |
| *Correctly following treatment pathways*  (Grant et al, 2012; Cassimatis, Kavanagh & Smith, 2014 – diabetes ; Jones et al, 2012) | - Confusion about initiating treatment - Lack of confidence re: self-titration automated BP uploads - pathway available on website - algorithm run by computer - verified by physician |
| *Frustration at ‘borderline’ readings*  (Jones et al, 2012) | - ‘Just’ amber readings trigger a titration – sometimes would not titrate in borderline cases but think carefully about it instead. |
| *Patients were concerned about being overmedicated*  (Halifax et al, 2007; Jones et al, 2012) | - Feeling that increased medication would be necessary for the rest of their lives |
| *HCP concerns about patient acceptance*  (Fairbrother et al, 2013 – chronic heart failure) | - Patients unwilling to accept more medication |
| **Engagement:** Patient and health professional experiences of DI (or other equivalent system) | |
| *Patients don’t want responsibility for care*  (Grant et al, 2012) | - Regular HCP appointments (does not replace usual visits) |
| *Increased HCP workload*  (Halifax et al, 2007; Bostock et al, 2010 ; Fairbrother et al, 2013 – chronic heart failure) | - Increasing clinic appointments was undesirable especially in non-urgent cases - Did not want to disrupt current workflow and systems - Concern about cost and clinical utility |
| *Increased HCP responsibility*  (Bostock et al, 2010 ; Fairbrother et al, 2013 – chronic heart failure; Hanley et al, 2013) | - Concern about being bound to act upon the information - Concerns about increased dependency and on clinicians and patient sick roles - Misalignment in expectations (i.e. patient perceived continuous and instant vs. actual level of monitoring) |
| Concerns about *detrimental effect on doctor-patient relationship*  (Grant et al, 2012) | - regular HCP appointments - medication treatment pathway designed by regular HCP |
| *Perceived problems of DI*  *(*Grant et al, 2012; Kerr et al, 20102 – heart disease) | - Too much effort - perceived as more difficult - less personal - less effective (than one-to-one discussions) - automated home monitoring device and pathway |
| *Confidentiality*  (Rahimpour et al (2008) | - concerns about the confidentiality of the system |
| **Acceptability:** Health professional confidence in the online system | |
| *Concern about reliability of home readings*  (Halifax et al, 2007) |  |
| *Problems integrating BP readings in to patient records*  *(Hanley et al 2013)* |  |
