# Supplementary material for: Intervention planning for a digital intervention for self-management of hypertension: a theory-, evidence- and person-based approach
Source: Implement Sci. 2017 Feb 23;12:25. doi: 10.1186/s13012-017-0553-4 (PMC5324312; doi:10.1186/s13012-017-0553-4)
Supplement: Additional file 5: — Excerpts from the quantitative review of evidence for intervention components used for hypertension self-management. (DOCX 18 kb) [file 13012_2017_553_MOESM5_ESM.docx]

**Additional file 5: Excerpts from the quantitative review of evidence for intervention components used for hypertension self-management**

| **Study** | **Self-monitoring schedules** | **Methods of BP feedback** | **Titration procedures** | **Type/ level of behavioural support** | **Length of intervention and follow-up** | **Control group** | **Reductions in blood pressure (SBP/ DBP)** | **Additional findings** |
| --- | --- | --- | --- | --- | --- | --- | --- | --- |
| McManus et al (2010) -  TASMINH-2: Tele-monitoring and self-management of hypertension (self-monitoring and self-titration of anti-hypertensive drugs according to a pre-planned titration schedule). | Training: patients received 2 training sessions from the research team where they were taught to self-monitor.  BP monitoring: BP readings were taken for the first week (7 days) each month.  Two recordings were made each morning with 5 minute intervals and the second recorded. | Blood pressure targets were set at <130/85 mmHg (or ≤130/75mmHg for patients with diabetes or CKD 3-5).  Colour coded feedback system:  Red – outside safety limits (> 200/100mmHg or systolic pressure <100mmHg)  Amber – above target but below safety limit  Green – below target but above lower safety limit  Red readings required the participant to contact his/her practice. | Two medication changes were agreed between patient and GP at the review meeting. Four or more ‘amber’ (above target) BP readings for two consecutive months resulted in the patient having to request the new prescription without consultation.  After two medication changes the patient had to attend a second medication review with the GP. | n/a | 12 months (follow-up at 6 and 12 months) | Post-randomisation review with the GP followed by usual care. | Mean of 2^nd^ and 3^rd^ clinic BP readings used as the primary outcome  3.7mmHg SBP reduction at 6 months  5.4mmHg SBP reduction at 12 months  No sig reduction in systolic BP over time between groups, nor anxiety or quality of life. | Incomplete cases at follow up were likelier to have higher deprivation scores, and a greater reduction in BP was observed for patients in lower deprivation scores.  Leg swelling was more common in intervention group (side effects).  The intervention was more effective by 0.24 and 0.12 quality adjusted life years (QALYs) gained per patient for men and women, respectively. The resultant incremental cost-effectiveness ratio for self-management was £1624 per QALY for men and £4923 per QALY for women. |
| Bosworth et al (2009) - Compared UC for hypertension with 3 groups: 1. Behavioural self-management, 2. Home monitoring and 3. Combination of the two (in this group the behavioural intervention was not adjusted according to BP readings). | Training: patients received BP monitor training from research assistants (and again at 6months if needed).  BP monitoring: BP readings were taken for 3 days per week (taken at the same time) and recorded in a log (returned to the study team every 2 months). | n/a | n/a | Behavioural self-management support was provided bi-weekly by a nurse (over the telephone), with each session covering different modules including perceived risk for hypertension, side-effects of anti-hypertensive medication, social support and lifestyle factors including DASH diet, weight loss, physical activity, salt and alcohol reduction. | 24 months (follow-up at 6, 12, 18 and 24 months) | Usual care: hypertension care from usual primary care provider. | Reduction in SBP compared to usual care at 12-months (95% CI)/ 24 months  Group 1: -1.6 (-3.9 to 0.7) / 0.6 (-2.2 to 3.4)  Group 2: -3.7 (-6.1 to -1.2)/ -0.6 (-3.6 to 2.3)  Group 3: -3.3 (-5.7 to -0.8)/ -3.9 (-6.9 to -0.9)  Reduction in DBP compared to usual care at 12-months (95% CI)/ 24 months  Group 1: -1.4 (-2.6 to 0.1)/ 0.4 9-1.1 to 1.9)  Group 2: -3.1 (-4.4 to -1.8)/ -1.2 (-2.9 to 0.4)  Group 3: -2.2 (-3.5 - -0.8)/ -2.2 (-3.8 to -0.6) | Combined intervention had greatest proportion of patients with BP control (at 24 months adjusted improvement compared to UC was 11%, 4.3% in behavioural management group and 7.6% in home monitoring group).  Interaction effects suggested that the BP monitoring and behavioural support enhanced one another over time – at 24 months systolic BP was only sig lower than UC in the combined group. |
| Green et al (2008) – 3 group RCT:  1. Usual care  2. Home blood pressure monitoring (E-BP)  3. E-BP plus pharmacist care management | Training: Patients were asked to demonstrate that they could use the monitor independently. Training was also given for using the website.  BP monitoring: BP readings were taken at least twice per week, with 2 readings taken at a time. | BP targets were set at <135/85 mmHg. | Patients in the third (pharmacist) condition sent BP readings every 2 weeks; pharmacists decided if medication changes should be made. | Pharmacists introduced patient action plan (instructions for BP monitoring, current meds, lifestyle goals, planned medication changes and follow-up plan). Support was provided every two weeks until BP was controlled (focussed on BP readings, medication concerns and lifestyle change progress). | 12 months | Patients were told their  BP was uncontrolled and to see their GP. All participants received a leaflet about hypertension. | Compared to usual care SBP at 12 months (mmHg, 95%CI):  Group 2 (monitoring): -6.0 (-8.5 to -3.5)  Group 3: -8.9 (-11.4 to 6.3)  Compared to usual care SBP at 12 months (mmHg, 95%CI):  Group 2 (monitoring): -6.0 (-8.5 to -3.5)  Group 3: -3.5 (-4.9 to -2.1)  1.8 times greater BP control with behavioural support and 1.2 times for monitoring only (compared with UC). | Incremental cost-effectiveness ratios (IC-ERs) calculated (change in costs relative to the change in outcomes measured).  BP monitoring and web-based pharmacist care improved BP control by 25% at a mean cost of $400 per patient, and an incremental cost of $16.65 per 1% increase of no of patients with BP control.  Pharmacist led programme – 1mmHg reduction in SBP was $65.29 ($114.82 for DBP). Increased cost in life expectancy was $1850 per year for women and $2220 per year for men. |
| Margolis et al (2013) – | Training: Patients received training from pharmacists during a 1-hour visit.  BP monitoring: Patients were asked to record at least 6 BP readings per week (3 taken in the morning and 3 in the evening) for the first 6 months. | Blood pressure targets were set at <135/85 mmHg (or ≤125/75mmHg for patients with diabetes or kidney disease). | Medication changes were made if <75% readings met blood pressure targets. | Pharmacist support was provided by telephone every 2 weeks until BP control was sustained for 6 weeks and then reduced to one per month for first 6 months (telephone contact was reduced to every 2 months during months 7-12). During pharmacist contact emphasized medication adherence and lifestyle changes. | 12 months (follow up at 6,12 and 18 months) | Usual care (consisting of pharmacist consultations and BP home measurement) | Compared to usual care SBP at 6, 12 & 18 months (mmHg): -10.7, -9.7, -6.6mmHg  Compared to usual care DBP at 6, 12 & 18 months (mmHg): -6, -5.1, -9.7 mmHg  BP control in intervention group: 6 months (71.8%), 12 months (71.2%), 18 months (71.8%)  Control group:  6 months (45.2%), 12 months (52.8%), 18 months (57.1%) | Significantly more medication escalation in the intervention group.  $1045 per patient for 12 month programme, estimate direct programme costs would be $1350per person.  Absolute difference in BP of 15% at 18 months compared to UC. |
