# Supplementary material for: Intervention planning for a digital intervention for self-management of hypertension: a theory-, evidence- and person-based approach
Source: Implement Sci. 2017 Feb 23;12:25. doi: 10.1186/s13012-017-0553-4 (PMC5324312; doi:10.1186/s13012-017-0553-4)
Supplement: Additional file 6: — Behavioural analysis of HOME BP using the Behaviour Change Wheel (BCW) and Normalisation Process Theory (NPT). (DOCX 26 kb) [file 13012_2017_553_MOESM6_ESM.docx]

**Additional file 6: Behavioural analysis of HOME BP using the Behaviour Change Wheel (BCW) and Normalisation Process Theory (NPT)**

| **Patient intervention components** | | | | | | | |
| --- | --- | --- | --- | --- | --- | --- | --- |
| Target Behaviour | Barriers/ *facilitator* to the target behaviour | Evidence for barrier/ facilitator/ intervention ingredient | Intervention ingredient  [location in HOME BP] | Target construct (BCW) | Intervention function (BCW) | BCT  (using 93 BCT taxonomy v1) | Target construct (NPT) |
| **Key behaviour: patient engagement with the intervention** | | | | | | | |
| Patient log on (following randomisation at baseline) | Forgetting | ***Martin et al., 2011;***  ***Huff et al., 2011*** | Patient email reminder Email to supporter (if patient not logged on 2 weeks after session 1 available)  [email/ Initial log-on] | Opportunity | Environmental restructuring | 7.1 Prompts/cues; | Cognitive participation (initiation) |
| Logging on to the website and entering readings | Limited time, competing priorities, forgetting  *Clear, feedback messages using traffic light system, focus on health status; knowing health status over time* | ***SMILE feasibility study; McManus et al, 2010 -TASMINH2; 2014, TASMIN-SR ; Ahern et al, 2012; Bostock et al., 2010; Halifax et al, 2007;Fairbrother et al, 2013; Rahimpour et al., 2008*** | Automated email prompts to remind to enter BP readings; Flexible bi-weekly data entry. Automated email reminders and nurse follow-up; traffic light system for comprehension of BP readings when log in (blue – low; green – target BP; amber – above target BP; red – high BP). Patient BP readings sent by email for patient records. Free text response option with the BP entry  [BP entry] | Opportunity | Environmental restructuring; | 7.1 Prompts/cues;  2.6 Biofeedback;  3.1 Social support | Collective action (interactional workability) |
| **Key behaviour: patient home blood pressure self-monitoring** | | | | | | | |
| Patient BP home monitoring | Willingness to self-monitor  *Concern about HTN* | ***Huff et al., 2011;***  ***Ahern et al., 2012; Halifax et al., 2007; Hanley et al., 2013; Anthony et al., 2012.*** | Building motivation for monitoring; health benefits of self-monitoring; motivational quiz outlining links between BP and other conditions  [Session 1] | Reflective Motivation | Education; Persuasion | 5.1 Inform of health consequences;  9.2 Pros and cons | Coherence (individual specification) |
| Patient BP home monitoring | Anxiety that home readings will not be as accurate as GP clinic readings  *Reliability and accuracy; concern about BP variability* | ***Agarwal et al., 2011; Bostock et al., 2011; Halifax et al., 2007; Fairbrother et al., 2013; Grant et al., 2012; Rahimpour et al., 2008*** | Information presented in session 1 reassuring that home monitoring is more accurate than clinic readings; it can lower BP readings and result in faster changes to medication.  [Session 1] | Reflective Motivation | Education | 5.1 Inform of health consequences; | Collective action (relational integration) |
| Patient BP home monitoring | Not having the skills/  confidence to monitor own BP at home | ***SMILE feasibility study; McManus et al, 2010; 2014; Watson et al., 2012; Green et al., 2008; Rahimpour et al., 2008*** | Teaching patient how to monitor their own BP  [Session 2] | Psychological Capability | Training;  Education | 4.1 Instruction on how to perform the behaviour;  6.1 Demonstration of the behaviour;  8.1 Behavioural practice/ rehearsal; | Collective action  (skill set workability) |
| Patient BP home monitoring | Not having the confidence to monitor BP alone  *Perceived value of HCP contact and support* | ***Green et al., 2008; Margolis et al., 2013; Hanley et al., 2013; Rahimpour et al., 2008; Haartman et al., 2007; Fairbrother et al., 2013*** | Reassurance there will be nurse support.  [Session 1]  Face to face nurse contact and email support (every 2 weeks until BP stable).  [Supportive emails] | Social opportunity | Environmental restructuring;  Enablement | 3.1 Social support | Collective action  (relational integration) |
| Patient to collect home BP monitor from practice | Limited time, competing priorities, forgetting | ***Martin et al., 2011; Huff et al., 201*** | Online notification and email prompt. Notify practice, practice lead and GP of randomisation to DI  [Session 1 and 2] | Physical Opportunity | Environmental restructuring | 7.1 Prompts/cues; | Cognitive participation (initiation) |
| Patient BP home monitoring and entry | Limited time, competing priorities, forgetting  *Free-text responses accompanying data entry* | ***SMILE feasibility study; Grant et al., 2012; Anhoj & Nielsen, 2004;***  ***Langstrup, 2008; Specialist clinician input – (IP&D meetings)*** | Flexible weekly monitoring to encourage habit formation; monitor twice a day (not 4 times); email prompts as reminders.  [BP entry] | Opportunity; Automatic motivation (habit formation) | Environmental restructuring;  Enablement | 2.4 Self-monitoring of outcome(s) of behaviour;  8.1 Behavioural practice/ rehearsal  8.3 Habit formation  7.1 Prompts/cues; | Collective action  (contextual integration) |
| **Key behaviour: patient medication adherence and titration** | | | | | | | |
| Patient adhering to medication titration | Unconvinced/ concerned about necessity of medication escalation | ***Okonofua et al; 2006; Lasserson et al., 2012; McManus et al, 2010 - TASMINH2; 2014 - TASMIN-SR; Huff et al., 2011; Gwadry-Sridhar et al., 2013; Benson & Britten, 2002; Svensson et al., 2000; Morrison et al., 2015.*** | Building motivation for medication adherence and titration (session 1 - motivation to avoid harm to future health and motivation to receive appropriate treatment) necessity/benefits and risks of BP medication; evidence that increasing BP medication does not increase side-effects.  [Session 1] | Reflective Motivation | Education | 5.1 Inform of health consequences; | Cognitive participation (legitimation);  Collective action (interactional workability) |
| Medication titration in borderline cases | Unconvinced/ concerned about necessity of medication escalation | ***Langstrup, 2008;***  ***Bostock et al., 2010; Jones et al., 2012; SMILE feasibility study.*** | Rationale for necessity of medication escalation provided in Session 1; will receive notification of medication change in writing from GP; opportunity to send GP message with BP readings.  [BP entry] | Reflective Motivation;  Social opportunity | Education; Persuasion;  Environmental restructuring; | 5.1 Inform of health consequences;  9.1 Credible Source  9.2 Pros and cons | Coherence (differentiation) |
| Entering correct blood pressure readings when second consecutive amber reading | Awareness that this will prompt the titration process | ***SMILE feasibility study.*** | Rationale for necessity of medication escalation provided in Session 1; Opportunity to send additional information to GP; BP machine recordings can be checked  [BP entry] | Reflective Motivation;  Opportunity | Education; Persuasion;  Environmental restructuring; | 5.1 Inform of health consequences;  9.1 Credible Source  9.2 Pros and cons | Collective action (interactional workability) |
| Making medication change without GP consultation | Patients may be anxious about titration, or doing it alone | ***Grant et al., 2012; Hartmann et al., 2007; Bostock et al., 2010;***  ***Jones et al., 2012;***  ***Figueiras et al., 2010;*** | Opportunity to send additional information to GP when entering readings, will receive notification of medication change in writing from GP; presenting medication change as ‘trial’; email support from nurse and face-to-face contact offered two weeks after titration. | Social opportunity | Environmental restructuring;  Enablement | 9.1 Credible Source  3.1 Social support | Coherence (individual specification) |
| Collecting new medication prescription | Limited time, competing priorities, forgetting | ***Grant et al., 2012;; Halifax et al., 2007*** | Email/ post notification of medication titration in writing from GP; nurse contact two weeks after titration | Opportunity | Environmental restructuring | 7.1 Prompts/cues;  3.1 Social support | Cognitive participation (initiation) |
| Adhering to new medication regime | Unconvinced/ concerned about necessity of medication escalation | ***Grant et al., 2012; Halifax et al., 2007; Bokhour et al., 2012; Benson & Britten, 2002;*** | Link back to rationale for medication escalation and information addressing concerns (minimal side effects, opportunity to trial medication); electronic support and encouragement from nurses, face-to-face support offered post-titration. | Reflective Motivation;  Social opportunity | Education; Persuasion;  Environmental restructuring;  Enablement | 5.1 Inform of health consequences;  7.1 Prompts/cues;  3.1 Social support; 9.1 Credible Source;  9.2 Pros and cons | Reflexive monitoring (reconfiguration) |
| **Key behaviour: patient lifestyle change** | | | | | | | |
| Adherence to lifestyle change modules | Unconvinced or unmotivated, lack of time. | ***Bennett et al., 2010; Hanley et al., 2013; Hyman et al., 2007; Haber et al., 2008.*** | Online lifestyle module describing the different lifestyle changes that people can make, provide guidance on selecting lifestyle changes (lifestyle changes optional) Nurse support provided. [Session 3] | Reflective Motivation | Education; Persuasion; | 5.1 Inform of health consequences; | Reflexive monitoring (reconfiguration) |
| **Health professional intervention components** | | | | | | | |
| **Key behaviour: health professional engagement with the intervention** | | | | | | | |
| Engaging with study | Concern that DI would increase workload or disrupt current workflow systems  *DI can overcome clinical inertia* | ***Halifax et al., 2007; Ahern et al., 2012; Santaschi et al., 2008; McManus et al., 2010; Green et al., 2008; Margolis et al., 2013.*** | Information outlining how the intervention is evidence-based, and will facilitate best practice, increase efficiency through automated processes (based on accurate home readings and reduce unnecessary consultations), with appropriately built-in communication and safety procedures. Information about the study procedures for participants (patient and health professionals). | Reflective motivation | Persuasion | 5.3 Information about the social and environmental consequences;  9.1 Credible source | Coherence (differentiation) |
| Completing online training | Limited time, competing priorities, forgetting | ***SMILE feasibility study.*** | Training completion monitored, automated reminders, follow-up by practice lead prior to recruitment.  [Prescriber training session and Supporter training session] | Opportunity | Environmental restructuring | 7.1 Prompts/cues | Collective action (skillset workability) |
| Follow-up PN and GPs who have not completed training | As above. | ***SMILE feasibility study.*** | Automated reminders/ email. Practice lead to also liaise with staff who have not completed the training. Study team to liaise with practice lead to ensure this is completed. | Opportunity | Environmental restructuring | 7.1 Prompts/cues;  2.1 Other(s) monitoring with awareness  2.2 Feedback on behaviour | Cognitive participation (initiation) |
| **Key behaviour: enacting medication titration procedures** | | | | | | | |
| Prescribing medication escalation appropriately | *Awareness of NICE and BHS prescribing guidelines* | ***SMILE feasibility study; Expert clinician input; Onysko et al, 2006.*** | Online prescribing algorithm provided to all HOME BP prescribers. Example scenarios will be provided (illustrating how to increase and change anti-hypertensive medication). Prescribers can access online medication titration records (or can record pre-planned changes in patient notes).  [Prescriber training session] | Psychological capability | Education | 4.1 Instruction on how to perform a behaviour  7.1 Prompts/cues;  8.2 Behaviour substitution | Coherence (communal specification) |
| Medication titration and escalation procedures | Not convinced about clinical appropriateness of escalating treatment | ***SMILE feasibility study;*** ***Wolf-Maier et al, 2007; Lawes et al, 2008, Waksh et al, 2006.*** | Evidence that clinical inertia is a problem for patients with hypertension, outlining the health related risks of uncontrolled hypertension and how this can be overcome with reliable home BP readings, and that these reading will be persuasive for patients.  [Prescriber training session] | Reflective motivation | Education; Persuasion | 5.1 Inform of health consequences; | Cognitive participation (enrolment) |
| Implementing intervention titration procedures | Unconvinced of feasibility in general practice | ***SMILE feasibility study; Hyman et al, 2012.*** | Provide evidence that HOME BP can overcome the problems in general practice by increased confidence in clinical decisions, as these are made in line with national guidelines and based on accurate BP readings (i.e. an average taken over two weeks over two months). Further evidence for reduced patient inertia, HOME BP will increase patient motivation for BP control, increase patient understanding about the necessity of medication and provide the skills and equipment to accurately self-monitor at home.  [Prescriber training session] | Opportunity | Environmental restructuring | 9.2 Pros and cons of implementing escalating treatment  5.1 Inform of health consequences;  12.2 restructuring the social environment  9.1 Credible source | Coherence (individual specification) |
| Implementing intervention prescription procedures | Limited time, competing priorities, forgetting | ***SMILE feasibility study;*** ***McManus et al, 2010; Green et al, 2008; Ahern et al, 2012; Grant et al, 2012.*** | Titration procedure to facilitate target behaviours automated reminders; providing a template email. Patient to be notified of medication change and HOME BP will ask the patient if they have been contacted by the practice.  [Prescriber training session] | Opportunity | Environmental restructuring | 7.1 Prompts/cues;  8.2 Behaviour substitution  2.7 feedback on outcome(s) of behaviour awareness | Collective action (skill set workability) |
| Prescribing escalating medication appropriately in borderline cases | Uncertain about clinical necessity | ***SMILE feasibility study;*** ***Halifax et al, 2007; Grant et al, 2012; Langstrup, 2008; Hyman et al, 2012*** | HOME BP will automatically recommend a medication titration when BP is above target for too long (automating the decision) based upon BP readings that are consistently above target.  [BP entry] | Motivation | Education; | 5.1 Inform of health consequences;  7.1 Prompts/cues | Cognitive participation (enrolment, legitimation) |
| Prescribing escalating medication appropriately in borderline or complex cases | Concern about patient acceptance, safety | ***SMILE feasibility study;*** ***Halifax et al, 2007; Grant et al, 2012*** | Provision of safeguards (patients given the opportunity to provide additional information at the BP entry if important; an offline procedure for very high or very low BP readings; patient and prescriber can revise or reject medication titration if appropriate). Automated online provision of persuasive advice to patients (including template letter to accompany prescription)  [BP entry] | Psychological capability;  Opportunity | Education; environmental restructuring | 5.1 Inform of health consequences;  7.1 Prompts/cues;  8.2 Behaviour substitution  1.5 review behavioural goals  1.6 Discrepancy between current behaviour and goal | Coherence/ cognitive participation (enrolment, legitimation) |
| **Key behaviour:** **behavioural support provision** | | | | | | | |
| Email contact providing encouragement to participants | Nurses unsure when to make contact;  *Nurse commitment* | ***SMILE feasibility study;*** ***Ahern et al, 2012; Green et al, 2008; Margolis et al, 2013; Uhlig et al, 2013; Bosworth et al, 2009*** | Supporter training will introduce the CARE (Congratulate, Ask, Reassure, Encourage) approach for providing behavioural support for the patient, Examples of how to implement CARE support provided for each of the key behavioural issues (i.e. supporting the patient to correctly use the BP monitor, not measuring blood pressure in support appointments, encouraging lifestyle change). HOME BP prompts to initiate contact, email scripts provided. Study team copied into email correspondence.  [Supporter training session] | Opportunity | Environmental restructuring | 7.1 Prompts/cues;  2.2 Feedback on behaviour | Collective action (skill set workability) |
| Providing face-to-face (or telephone) support sessions with patients | Nurse forgetting | ***As above*** | DI prompts to initiate contact sent to usual email address. Patients will be reminded when these are available at key points, the patient will initiate these, and can request them at different points. | Opportunity | Environmental restructuring | 7.1 Prompts/cues; | Collective action (skill set workability) |
