# Supplementary material for: Intervention planning for a digital intervention for self-management of hypertension: a theory-, evidence- and person-based approach
Source: Implement Sci. 2017 Feb 23;12:25. doi: 10.1186/s13012-017-0553-4 (PMC5324312; doi:10.1186/s13012-017-0553-4)
Supplement: Additional file 7 — Applying the NPT and BCW theoretical frameworks to the HOME BP intervention content: an analysis of patient intervention components for each NPT and BCW construct. (DOCX 19 kb) [file 13012_2017_553_MOESM7_ESM.docx]

**Additional file 7: Applying the NPT and BCW theoretical frameworks to the HOME BP intervention content: an analysis of patient intervention components for each NPT and BCW construct**

| **NPT construct** | **Examples in HOME BP context** | **Patient intervention components** (location in the intervention) |
| --- | --- | --- |
| **Coherence (sense-making work)** | | |
| Differentiation | Understanding and differentiating between aspects of the illness & treatment – for example, when titration is necessary and when it is not | Rationale for necessity of medication escalation provided in Session 1 (link back to this information at point of titration). |
| Communal specification | Gaining info about the condition and its management with the help of others | All HOME BP participants will be given access to the patient.co.uk information sheet for hypertension. |
| Individual specification | Gathering own info about condition, or developing own understanding | Session 1 - Building motivation for monitoring and titration; information on side effects (necessity and risks); motivational quiz; information to reassure patients that monitoring at home can be more accurate than clinic readings.  Notification of medication change will be received in writing from GP (re-specifying the medication regime following titration). |
| Internalization | Understanding the implications of treatment and when titration is necessary | Session 1 – Motivating patients to avoid harm and to seek appropriate treatment; necessity and risks of medication titration |
| **Cognitive Participation (relationship work)** | | |
| Enrolment | Engaging with others (family, HCP) to enable them to support BP management | HOME BP behavioural support will be offered approx. 2 weeks after Session 2 (face-to-face) with other face-to-face support offered at essential times (e.g. following the introduction of the optional lifestyle change modules) and email support in interim periods |
| Activation | Arranging help from HCPs or others |  |
| Initiation | Organisational aspects of behaviour involved in management of blood pressure | Session 1 – Persuasive evidence outlining why medication adherence is the right thing to do. Electronic prompts to collect BP monitor (to enable home self-monitoring); prompts to remind when to self-monitor and enter readings (prompted by emails); collecting new prescription/medication following titration process. |
| Legitimation | Seeking reassurance from others about appropriateness of management plans | All patients enrolled in HOME BP will have a baseline medication review with the HOME BP prescriber; in the intervention group only this will involve agreement of the medication titration process. |
| **Collective Action (enacting work)** | | |
| Skill set workability | Developing the skills and routine to self-monitor | Patients will be trained in the correct procedure for BP monitoring during Session 2 – this will include undertaking practice readings and outlining the information regarding how often they will need to monitor and when they will need to enter readings into the website. Face-to-face nurse support is offered following this to provide support with any monitoring problems. A (flexible) patient home self-monitoring routine will be established (7 days readings taken every 4 weeks, or every 8 week once controlled). |
| Contextual integration | Having the right social and financial resources; integration of the illness in to social situation | Monitoring will be flexible to encourage habit formation, and email prompts sent as reminders to facilitate integration of BP monitoring within daily life. |
| Interactional workability | Taking medications, medication side-effects, engaging with DI, lifestyle changes – integrating all of these in to everyday life. | Patients will be able to flexibly log on to HOME BP, and enter the last day of their 7^th^ reading. Information collected at BP entry to understand patient reasons for non-titration (if applicable) and behavioural support for lifestyle changes. |
| Relational integration | Developing relationships with HCPs  Building confidence in the system and in relationships with HCPs | Information provided in Session 1 to reassure patients that home readings are accurate (building confidence in the system). Developing confidence in and relationships with practice nurses offering support for monitoring, medication titration and lifestyle change modules. |
| **Reflexive monitoring (appraisal work)** | | |
| Reconfiguration | Altering a set routine (such as medication escalation) when required | Adopting new medication regime (following titration); engaging with new lifestyle change modules. |
| Communal appraisal | Discussing or altering current management plans with others (family, HCPs) | HOME BP medication titration plans will be agreed between patients and HOME BP prescribers; patients will be able to request support throughout the study. |
| Individual appraisal | Patient chooses to continue with self-monitoring or treatment escalation regime | Patients will receive BP feedback from HOME BP each month, after 7 BP entries have been entered. This will provide objective evidence for medication changes (and continued) self-monitoring where necessary. At a recommended medication change, patients will be able to provide the prescriber with additional information – the prescriber may then decide to overrule the medication titration. Patients can also decide against initiating a medication change (but will be asked to provide information for their reasons to do so). |
| Systematization | Ways to keep up with newly available treatments |  |

| **BCW Intervention function** | **Examples in HOME BP context** | **Patient intervention components** (location in the intervention)  *No intervention components were addressing this intervention function* |
| --- | --- | --- |
| Education | Increasing knowledge or understanding of hypertension, self-monitoring and anti-hypertensive medication | Patients are provided with information in Session 1 relating to the benefits of home self-monitoring and medication escalation for blood pressure control. Information about the correct procedure for home self-monitoring is provided within Session 2. Further education about lifestyle changes for reducing blood pressure are provided in Session 3. |
| Persuasion | Inducing positive feelings about hypertension self-monitoring, medication adherence and lifestyle change to increase self-management | Motivational quiz to highlight the links between hypertension and other health conditions (Session 1). Persuasive evidence provided to increase motivation for monitoring and medication escalation procedures involved in HOME BP (Session 1). Optional, self-selected lifestyle changes available (Session 3) and reassurance that support will be provided for key behaviours (self-monitoring and lifestyle change). |
| *Incentivisation* | *Creating expectation of reward* | *No examples of incentivisation are included in HOME BP* |
| *Coercion* | *Creating expectation of punishment of cost* | *No examples of coercion are included in HOME BP* |
| Training | Providing patients with the skills needed to undertake the target behaviours (particularly correctly self-monitoring blood pressure). | Patients are provided with blood pressure self-monitoring training and a practice BP entry to ensure they are confident and competent to self-monitor (Session 2). Patients can send practice readings to the HOME BP supporter, and discuss if they are having problems undertaking this. |
| Enablement | Facilitating patient self-management of hypertension by increasing capability and opportunity and addressing potential barriers | Monitoring schedules will be routine, but flexible, with email prompts sent to patients as reminders. Patients are reassured that support will be available to them throughout the HOME BP study, which will include face-to-face support at crucial behaviour change points and monthly email support (until BP is stable). Patients will also be able to request support at any time and will be given the opportunity to provide additional information to the prescriber when a medication change is recommended. |
| Modelling | *Provide an example for people to aspire to or imitate* | *No examples of modelling are included in HOME BP* |
| Environmental restructuring | Changing the physical or social context to facilitate self-monitoring, medication adherence or lifestyle change. | Patients will be sent email prompts to engage in target behaviours (log on, collect BP monitor, collect prescription, monitor BP, enter BP readings), which will be followed up by the HOME BP supporter when behaviour not enacted within a specified period. HOME BP will provide an opportunity to undertake monthly blood pressure self-monitoring and receive instant blood pressure feedback; which will also be sent by email to the patient for them to refer back to if needed. Patients will be offered and may request behavioural support throughout the study, and will have the opportunity to include further information to accompany the blood pressure readings to the prescriber in the case of a recommended medication change. |
| *Restrictions* | *Using rules to reduce the opportunity to engage in the target behaviour (or to increase the target behaviour by reducing the opportunity to engage in competing behaviours).* | *No examples of restriction are included in HOME BP* |
