# Supplementary material for: Intervention planning for a digital intervention for self-management of hypertension: a theory-, evidence- and person-based approach
Source: Implement Sci. 2017 Feb 23;12:25. doi: 10.1186/s13012-017-0553-4 (PMC5324312; doi:10.1186/s13012-017-0553-4)
Supplement: Additional file 8: — Excerpts from the key findings arising from qualitative and quantitative literature searches to identify studies examining the potential determinants of key HOME BP patient behaviours. (DOCX 17 kb) [file 13012_2017_553_MOESM8_ESM.docx]

**Additional file 8: Excerpts from the key findings arising from qualitative and quantitative literature searches to identify studies examining the potential determinants of key HOME BP patient behaviours**

| **Study** | **Focus of the study** | **Main study findings** | **Summary of potential determinants for HOME BP** | **Potential behaviour change theories for logic model** |
| --- | --- | --- | --- | --- |
| Bokhour et al (2012)^1^ | Hypertension self-management (including medication adherence and lifestyle change) | - Hypertension self-management was affected by patient explanatory models with regard to the cause, course and symptoms of hypertension and treatment beliefs, as well as social context factors. - Cause: patients describe hypertension and temporary increases in BP as separate, and described engaging in behaviours to address the latter rather than the former (i.e. BP increases as a result of stress, pain or over-exertion). - Course: some patients viewed hypertension as something intermittent that only required focus when BP was too high (not a constant problem) - Symptoms: some patients described no symptoms associated with hypertension (and so consequently felt little need to adhere to medication) whilst others described being able to feel when BP was high (and consequently only adhering when perceived symptoms associated with high BP). - Treatment: beliefs about treatment were linked to cause and symptom beliefs, for example, beliefs about medication harm led to reliance on relaxation more often. - Social context: factors such as a lack of routine, important medication side effects and lifestyle factors (such as drinking alcohol) were | - Accurate causal beliefs about hypertension - Coherent understanding of hypertension and high blood pressure - Dissociating perceived symptoms from patient self-management behaviours (so that treatment is linked to evidence rather than heuristics) - Informing participants about the risks associated with anti-hypertensive medication - Facilitating self-management (including medication adherence and lifestyle change) within the everyday context | *Illness beliefs (CSM)*  *Treatment beliefs (CSM/ SCT)*  *Lifestyle behaviour change (BCW/ NPT)* |
| Figueiras et al (2010)^2^ | Hypertension illness perceptions and medication selection | - Patient’s perceived medication to be necessary and showed moderate concern about medicines. - Medication necessity beliefs associated with negative consequences, chronic timeline, greater personal and treatment control, lots of perceived symptoms, concern and negative emotional representations. - Medication concern associated with negative consequences, chronic timeline, and high levels of perceived symptoms, concern and negative emotional representations. - Identified three clusters of hypertension beliefs:  1. Serious consequences, chronic timeline, strong personal & treatment control, very high illness concern, negative emotional representations, high illness coherence 2. In comparison to cluster 1 – less negative consequences, identity, concern and emotional representations 3. Less strong illness identity than cluster 1 and 2, positive beliefs about consequences and timeline, less personal and treatment control, lower concern and comprehensibility and more positive emotional representations.   These clusters were associated with medication selection behaviours. | - Medication beliefs are linked to patient understanding about hypertension and its treatment - Different illness models for hypertension which seems to be associated with (potentially) with different medication acceptance. - Changes in understanding of hypertension may be an important mediator in HOME BP | *Illness beliefs (CSM)*  *Treatment beliefs (CSM/ SCT)* |
| Brailsford & Green (unpublished thesis)^1^ | Medication intensification | - Patients reported that they would not refuse medication increases but would want to know what it was necessary - If patients saw medication as one ‘dose’ would accept this (even if it included multiple medications) - Patients may hold beliefs that are inconsistent with medical models – and relationships with HCP’s can influence IPs and acceptance - Patients saw medication as a ‘necessary evil’ | - Important to evidence why medication increases are necessary for patient acceptance | *Treatment beliefs (CSM/ SCT)* |
| Benson & Britten (2002) ^1^ | Beliefs about medication | - Patient decision making for taking anti-hypertensive drugs were organised around three themes:  1. Reservations about drugs generally – patients reported beliefs that drugs are unnatural or unsafe; highlighted the importance of others’ or own experiences of adverse experiences; beliefs about medications being prescribed too readily; and crucially about medication signifying ill health. 2. Reservations about anti-hypertensive medication specifically – patients reported exploring potential alternatives, and discussed issues surrounding the necessity of anti-hypertensive medication and possibility of long-term or hidden risks. 3. Reasons to take anti-hypertensive medication – patients reported that seeing improvements after taking anti-hypertensive medication encouraged medication adherence; perceived benefits of taking medication benefits (such as **feeling better**, gaining peace of mind or other good outcomes) were important; some reported pragmatic considerations (such as no other practical solutions to controlling BP); positive, encouraging experiences with health professionals were key.  - Most patients reported balancing the reservations with the reasons to take anti-hypertensive medications. | - General concerns about medications - Specific beliefs about the necessity, risks, and benefits of anti-hypertensive medication - Feeling better was the main reason to take anti-hypertensive medication - Greatest reservation around taking medication linked to perceived ill health | *Treatment beliefs (CSM)* |
| Ross et al (2004)^2^ | Medication adherence | - Patient medication adherence was influenced by medication necessity beliefs, older age, female gender and cure beliefs. - Concerns about anti-hypertensive medications, higher emotional responses, lower consequence perceptions were associated with less medication compliance - Patient age, specific-necessity beliefs, emotional representations   and personal control perception were most predictive  of adherence   - Medication necessity beliefs predicted by age, number of medications, chronic timeline and consequence/ cure - Medication concern beliefs were predicted by general harm beliefs, general overuse, emotional representations, age and perceptions of consequences. - No relationship was found between cause perceptions and compliance | - Medication adherence and non-adherence are associated with different illness and medication beliefs - Beliefs about hypertension and anti-hypertensive medication are interlinked - Beliefs about the necessity of medication are important for medication adherence - Addressing patient concerns about medications is important | *Illness beliefs (CSM)*  *Treatment beliefs (CSM)* |

Note. Extended Common Sense Model (CSM); Social Cognitive theory (SCT); ^1^ Indicates qualitative study findings; ^2^ Indicates quantitative study findings^.^ The Summary of potential determinants for HOME BP are referred to in Figure 4 as ‘proposed mediating variables’
